# Clothes, Sensory Experiences and Autism: Is Wearing the Right Fabric Important?

**DOI:** 10.1007/s10803-021-05140-3

**Published:** 2021-07-21

**Authors:** Chrysovalanto Kyriacou, Rachel Forrester-Jones, Paraskevi Triantafyllopoulou

**Affiliations:** 1 Tizard Centre, School of Social Policy, Sociology and Social Research, University of Kent, Canterbury, Kent UK; 2 Department of Social and Policy Sciences, Centre of Analysis of Social Policy, University of Bath, Bath, UK

**Keywords:** Autism, Sensory differences, Tactile defensiveness, Fabrics, Clothes

## Abstract

Tactile defensiveness in autistic individuals is the least investigated sensory modality. The current multi-component, explorative study aimed to understand the experiences of ten autistic adults regarding tactile defensiveness and fabrics, using semi-structured, one-to-one interviews. Participants were asked to discuss the effects of seven provided samples of fabrics and were also asked to bring their ‘favourite’ fabric (s) and express their thoughts about their choices. Using Interpretative Phenomenological Analysis and Content Analysis, the findings showed that some fabrics can impact individuals’ reported wellbeing. Participants’ experiences with several stimuli appeared to have helped them implement coping strategies. By understanding tactile defensiveness, society could move towards increasing autism-friendly approaches with appropriate fabrics. Recommendations for future research, policy and practice are also discussed.

**Supplementary Information:**

The online version contains supplementary material available at 10.1007/s10803-021-05140-3.

## Introduction

Autistic individuals have persistent differences in verbal and non-verbal communication, social interaction, restricted and repetitive patterns of behavior and interests, as well as uncommon responses to sensory stimuli from hyper-responsiveness to hypo-responsiveness (American Psychiatric Association DSM-5, 2013). Leekam et al. (2007) concluded that more than 90% of autistic children and adults had sensory responsiveness interfering with their everyday lives, and their symptoms were persistent across all age groups and IQ levels (Baranek et al., 1997; Ben-Sasson et al., 2009; Forrester Jones and Broadhurst, 2007; Harrison & Hare, 2004; Kern et al., 2007).

Autistic individuals react to sensory stimuli with a behavior that is not comparative to the grade and nature of the sensory stimulation (Baranek et al., 2006; Lane et al., 2010). Hypo-responsiveness refers to the lack of reaction to environmental or bodily sensory stimuli (Tomcheck, and Dunn, 2007). Hyper-responsiveness, is characterized as an overload of sensory stimuli (Frith, 1991; Kanner, 1943). As a result, autistic individuals may show sensory soothing behaviors that function to repeat or strengthen sensory experiences (Ben-Sasson et al., 2009; Damiano et al., 2018; Kirby et al., 2015). Hypo-responsiveness can occur distinctively or alongside sensory soothing behaviors (Dunn, 2007; Lane et al., 2011). Sensory soothing behaviors are linked to high neurological threshold, meaning that an individual requires intense sensory stimuli to produce a reaction that can be soothing (Dunn, 2007). In contrast, lower neurological threshold are linked to behaviors that reflect an avoidance to certain types of sensory input that others find innocuous (Butera et al., 2020; Dunn, 2007).

Through functional magnetic resonance imaging (*f*MRI) Green et al. (2015) found that autistic individuals experienced significantly more symptoms of hyper-responsiveness than neurotypical and developmentally delayed individuals. Several studies found significant differences between autistic and control groups (Baranek et al., 2006; Crane et al., 2009; Tavassoli et al., 2014) in a range of sensory modalities, like gustatory (Tavassoli & Baron-Cohen, 2012); olfactory/smell (Bennetto et al., 2007), auditory/hearing (Haesen et al., 2011), visual (Simmons et al., 2009) and tactile (Cascio et al., 2008). Autistic individuals experience touch dysfunction more often compared to the other sensory modalities (Kern et al., 2006); however it is still the least investigated compared to the extensive research on the other sensory modalities (Baranek, et al., 2006).

Tactile defensiveness is the hypo-responsiveness and hyper-responsiveness to direct touch stimuli (Baranek et al., 1997). Many autistic individuals experience an unusual anticipation of being touched, wearing specific clothes or from tags and labels on clothes (Kern et al., 2006). Autobiographical reports from autistic individuals explained that their uncommon sensory experiences were sometimes devastating and could act as incentives for social withdrawal (Grandin, 1992; Markram & Markram, 2010; Cosbey et al., 2010).

Despite being able to avoid unpleasant textures in the environment (Robertson & Simmons, 2015), autistic individuals experience emotional distress and anxiety when in contact with specific unpleasant stimuli (South & Rodgers, 2017). These sensory experiences may hinder autistic people’s educational development as they have been found to compromise their ability to concentrate in a classroom (Howe & Stagg, 2016). Autistic individuals have also reported that their sensory responsiveness and emotional distress are being part of a vicious cycle comprised of stress and increased responsivity (Smith & Sharp, 2013).

On the other hand, tactile modality is most commonly found to produce pleasurable experiences when it is perceived to be more controllable (Jones et al., 2003; Robertson & Simmons, 2015). In this respect, Autistic individuals have reported enjoyment when touching woolen fabrics, heavy blankets and rubbery objects (Ashburner et al., 2013). Some autistic individuals have also reported being able to control such pleasurable tactile experiences i.e. touching cold, even surfaces; using these textures as coping strategies to relax (Robertson & Simmons, 2015).

Despite discussion regarding the psychological, physical and social impact of tactile defensiveness, the majority of previous studies are based on proxy reports and self-report quantitative assessment tools (Ben-Sasson et al., 2009; Brown & Dunn, 2002). Since the empirical dimensions of tactile defensiveness, including how individuals experience different textures and fabrics in their daily environment, how they use and choose fabrics, and how tactile defensiveness affects their everyday lives has not previously been explored, qualitative methods were deemed the most appropriate to explore the complexities of individuals’ experiences. In this paper, following the findings of Kenny et al., (2016) as well as public and social media discourse, we use identify first language (i.e. autistic person) rather than person first language (i.e. person with autism), since the autistic community consider being autistic as part of their identify (Waldock and Forrester-Jones, 2019; Sinclair, 2013). This also fits with our qualitative approach that respects and accepts autistic voices as key to investigation autistic lives experiences.

The aim of the study was therefore to: (a) explore the importance of different type/texture of fabrics to autistic people; (b) investigate the effects of different fabrics have on the everyday lives of autistic people; and (c) understand if the color and texture of fabrics influences their experience. The objective was to increase awareness of tactile defensiveness and understand the impact of fabrics on the participant’s everyday lives, thereby adding to the very small body of qualitative research that focuses on the experiences of autistic individuals in regards to tactile defensiveness (Appendix Table 1).

## Research Methods

Interpretative Phenomenological Analysis (IPA) was used to explore autistic individuals’ subjective experiences of fabrics. IPA has three key philosophical approaches: ‘Phenomenology’, ‘Hermeneutics’ and ‘Idiography’ (Smith et al., 2009). ‘Phenomenology’ is a philosophical method to the understanding of people’s subjective experiences. ‘Hermeneutics’ refers to the active connection between the researchers’ interpretation of the participants’ experiences, whilst recognizing the effect of their own personal perception on the findings (double-hermeneutic) (Smith et al., 2009). ‘Idiography’ refers to the commitment of the researcher to collect in-depth data to understand these experiences from the participants’ perspective. IPA differs from other qualitative methods (such as thematic analysis and participant observation) in that its focus is on the expressed experiences of individuals of specific social phenomena (in this case the impact of different fabrics on their everyday functioning). IPA allows each individual to voice their own particular experience and for themes that are important to the group to emerge and take center stage in the research. This differs from quantitative methods that seek answers to a priori hypotheses.

Participants’ perceptions of seven samples of fabrics was explored and subjected to content analysis. Particular fabrics were chosen for the study based on previous enquiries within the local autistic community as to fabrics most commonly encountered (See Appendix Table 2). Through systemic organization and quantification of coding and identifying patterns, content analysis offers understanding of the complex models of human perception (Mayring, 2004).

### Design

The current study design was explorative, using semi-structured, in-depth, one-to-one interviews that allowed responses that linked to the participant’s subjective tactile experiences. Participants were provided with seven samples of fabrics (see Appendix Table 2) and were asked to explain the effects that these may have had on their everyday lives. They were also asked to bring their own favorite fabric to the interview and to share their feelings about the fabric. This multi-activity component of the design aimed to provide a better understanding of participants’ experience of tactile defensiveness.

### Participants and Settings

Due to the nature of the study, a purposive sampling approach was utilized. Participants were adults (above the age of 18) with a diagnosis of autism. The chosen sample included adults, since approximately 90% of the autistic adult population have been reported as having sensory responsiveness that interfere with their everyday lives (Leekam et al. 2007). University students were included because they were a readily available group.

Ten participants were interviewed (seven females; three males). The ideal number of participants in IPA is six to eight (Smith et al. 2009). Participants were all university students at different stages of their education (seven undergraduates; three postgraduates) and were recruited via gatekeepers including the university’s support services as well as the university’s Autism Group on Facebook via on-line advertisements. All participants confirmed their autism diagnosis prior to taking part in the study.

### Materials and Measures

Interview questions (Appendix Table 1) were not extracted from previous studies as there were no studies that have explored this subject area, therefore, the authors followed Smith et al. (2009) guidance on producing interview questions. The seven samples of fabrics used (satin, denim, hession, cotton, polyester, wool and spandex) (See Appendix Table 2) were chosen on the basis of previous qualitative descriptions of fabrics that have shown to affect autistic individuals (Ashburner et al., 2013; Blakemore et al., 2006; Cascio et al., 2016). Participants were also asked to bring their favorite fabric with them to the interview session to allow an exploration of subjectively positively experienced fabrics. The semi-structured open-ended informal interview schedule was designed to provide a calm interface with the participants and to facilitate their individual experiences (Smith et al., 2009). The interviews lasted 25 to 60 min and all participants gave their informed consent for the interview to be audio-recorded (see Appendix 1 for interview questions and pictures of the 7 fabrics) (Appendix Tables 1 and 2).

### Procedure

From the third question onwards, questions (Appendix 2) became more specific (semi-structured); yet still open-ended (See Appendix Table 1). On question 6 (See Appendix Table 1), participants were asked to touch each of the seven samples of fabrics (See Appendix Table 2) and describe their perceptions and how these might have affected them in their everyday life. The final question (See Appendix Table 1) asked participants to present their favorite fabric. They were then asked to explain the reasons behind their choice, and the usefulness/value of the specific fabric(s) for them. Throughout the interview, the researcher gave the participants regular feedback regarding her own interpretations to check if they were in line with the participant’s perceptions (Smith et al., 2009). The researcher debriefed the participants before leaving the interview room.

### Ethical Approval

The current study received a favorable ethical opinion from the University of Kent Tizard Ethics Committee, (Reference: 29,032,017). Informed consent in writing was obtained from all participants included in the study.

Potential participants were provided with an information sheet and consent form. The first researcher conducted the interviews and used a designated room at the University for the Interviews to take place. A £5 voucher was offered to all participants as a token of appreciation for their time. Consent was re-established prior to the start of the interview and the interview started by asking two open-ended questions to encourage the participants to talk in depth about their experiences (Smith et al., 2009). Participants were informed that they had the right to refuse to touch the fabrics, therefore if participants did not wish to touch the fabric(s) the researcher would advise them to respond as regards to their past experience with the specific fabric(s). No participants withdrew from the study and no distress was documented or reported to the researcher during, or after the interview.

Pseudonyms were used in the analysis of the data to protect the participants’ identities and data were treated in accordance to GDPR regulation.

### Analysis

#### Interpretative Phenomenological Analysis (IPA)

Once all of the audio-recorded interviews were transcribed, an idiographic approach was taken whereby, the first author read and re-read the transcripts to allow an active hermeneutic engagement with the data (Smith et al., 2009). Next, the researcher produced a broad set of codes which were compared to the second author’s independent codes of the same transcripts. This allowed for coding reliability. Discussions between the two authors let to coding-saturation. Patterns of similarity across the data took place until final themes began to emerge, which were then relabeled and reorganized.

#### Content Analysis

During the interview, the researcher requested that participants to touch each of the seven fabrics and explain their perceptions of each fabric, the effect each would have if they should encounter them in their everyday lives, and their fabric preferences. Content Analysis was then used to analyze the participant’s interpretations and experiences of the seven samples of fabrics. The subjective qualitative data obtained was coded and quantified into categories of liked/disliked and reasons (See Appendix Table 3).

### Reliability

The research team recognize the importance of evaluating qualitative research according to the criteria suitable for it. Therefore Guba’s (1981) four criteria of trustworthiness (Credibility-internal validity, Transferability-generalizability, Dependability-reliability and Confirmability-objectivity) were used to measure reliability in the current study.

Credibility was achieved as the questions, setting and analysis were all completed in the same way for all participants. Credibility and triangulation was strengthened through the use of one-to-one interviews, the displayed fabrics and by asking participants to bring their favorite fabric. Transferability and dependability are the hardest and most debatable aspects of qualitative research because the relationship between the researcher and the participants (Appendix 3) tends to be unique and thus, difficult to replicate (Gomm et al., 2000). Dependability/Reliability was ensured through the use of mini-audits, and inter-reliability of the codes and themes with the study’s second author (Smith et al., 2009). All scripts were reviewed by two of the authors and there was agreement on all themes and codes. Mitigation against any disagreements was reached, via the used of the principles of saturation. Confirmability was increased through the transparent details provided in the current study.

## Results

### Findings

**Fig. 1:**
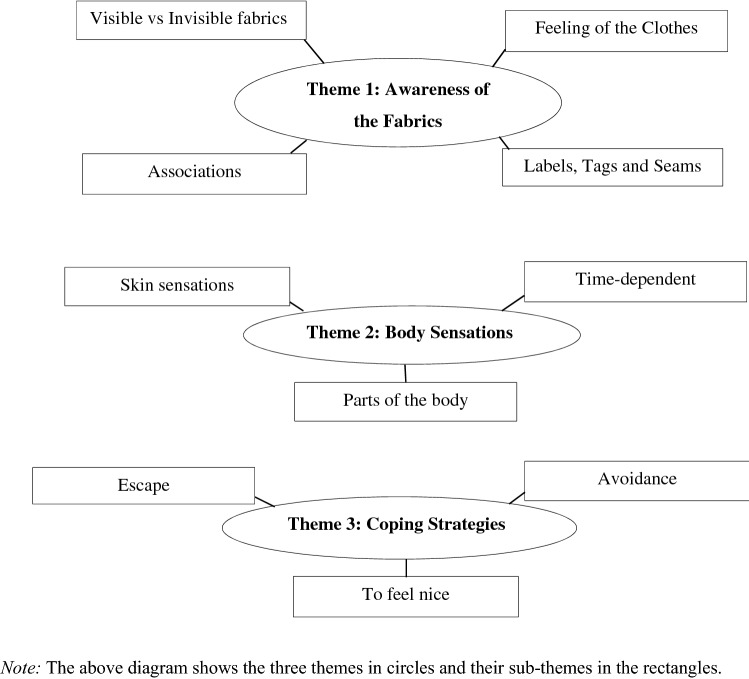
Diagram of Themes and Sub Themes. The above diagram shows the three themes in circles and their sub-themes in the rectangles

The themes presented below are (Appendix 4)supplemented by substantive anonymous quotes selected as the most descriptive, and the ones that expressed and represented the purpose of the themes. After conducting the IPA analytic steps proposed by Smith et al. (2009), there were three dominant themes that were extracted from the data: Awareness of the Fabrics, Body Sensations, and Coping Strategies. An illustration of the themes and subthemes can be seen in Fig. 1 below. The Awareness of the Fabrics theme, had four sub-themes: Visible vs Invisible fabrics, Feeling of the Clothes, Labels, Tags and Seams and Associations. The Body Sensations theme had three sub-themes: Time-Dependent, Parts of the body, and Skin Sensations. Lastly, the Coping Strategies theme consisted of three sub-themes: Avoidance, Escape, and To feel nice. Side effects of what the participants were experiencing were incorporated into all of the above themes, since these were interlinked (Appendix Fig. 1).

### Theme 1: Awareness of the Fabrics

This theme was related to fabrics which exist within the everyday living environments of participants, including, clothes as exemplified by Participant 8 (p.1, 20):

“I am constantly aware to a large extent on what I am wearing”.

Participants stressed the importance of both fabrics in their surroundings and the ones they were in physical contact with, since such encounters affect their wellbeing. This theme therefore encompassed four sub-themes: Visible vs Invisible fabrics; Restrictiveness; Labels, Tags, and Seams; Associations.

#### Visible vs Invisible Fabrics

This sub-theme related to fabrics and textures that were in the participants’ everyday lives and surroundings, which could either be seen, or which were attention-grabbing for them. Participants mentioned being concerned about irritating furnishings used in public spaces.

“I guess sometimes the library. Like the chairs are kind of itchy” (Participant 1).

“Schools should not use that horrible carpet” (Participant 2).

“I went on the bus earlier and it was actually quite itchy” (Participant 6).

Some expressed aversion towards noisy fabrics and textures, even if they were not visibly discomforting.

*“*you could hear the motion. I don’t like the sound of anything going over this material because it makes my head feel a little weird” (Participant 7).

“…when you rub it you can hear that noise which annoys me…” (Participant 2).

Other participants expressed negative side effects of their encounters with unwanted fabrics in the environment.

“I am always aware of everything, but I don’t want to feel that uncomfortable” (Participant 8).

“So I have to make sure I don’t touch the seats because when I get agitated can take over” (Participant 6).

On an optimistic note, positive feelings were expressed when they encountered positive textures in their environment.

“It’s really fascinating when you find something soft…” (Participant 1).

“So there are a lot of textures in the environment that could also help to decrease stress. Sometimes walking on grass in bare feet can be nice” (Participant 6).

#### Feeling of the Clothes

This sub-theme related to how participants felt being *inside* their clothes. Whilst wearing the right clothes in terms of how they felt was expressed as an important factor in their everyday lives. All of the participants reported that not being aware of the fabric was often an indication of their preference or feeling comfortable.

“So it’s good when you feel that something is not there because it means it’s comfortable, you know” (Participant 5).

“It’s really soft and I can’t really feel it because it makes me feel like I am naked” (Participant 7).

Most of the participants expressed that they preferred flexible clothes (or clothes that they could easily move around in) instead of restrictive and tight ones, having been experienced as uncomfortable in the past.

“…the tightness of it can be overwhelming” (Participant 10).

“I am kind of fuzzy you know when they’ve (socks) got that hem on the top because it feels a little suffocating” (Participant 3).

“I like cotton because they let you like, breathe and things” (Participant 8).

A high number of participants reported some of the side effects of having bad experiences with certain fabrics. These were mostly feelings of stress, distraction, and confusion.

“I’d gradually get more and more agitated and the more agitated I get, the more stressed I get and the more I move the less clearly you think because it goes round and round in circles.” (Participant 2).

“you can’t concentrate. You lose focus.” (Participant 6).

Participants also expressed their preference towards soft clothing, and how nice these made them feel.

“The softer the better usually. My go-to.” (Participant 9).

“…there is nothing that could irritate me because it’s so soft.” (Participant 6).

Regarding the color of the fabrics, the majority preferred muted colors, instead of patterned fabrics.

“…generally I choose quite plain colors” (Participant 10).

“…I do prefer things that are a bit more muted and not so obvious…” (Participant 2).

Many participants stated that being comfortable was much more important than the appearance of the fabric.

“I care more about the quality of the clothes I am wearing, and if they look nice that’s a plus.” (Participant 4).

“For me it’s more about how it feels than how it looks.” (Participant 6).

#### Labels, Tags, and Seams

The participants of the study disclosed their unique sensory awareness. The current sub-theme was related to the fact that the majority of participants reported their dissatisfaction towards the presence of, and contact with labels, tags and seams on their clothes.

“…it’s usually labels that affect me the most” (Participant 10).

“The labels are just there to make you feel bad” (Participant 1).

Some participants commented that the location of the labels, tags and seams placed within clothes, had made them feel uncomfortable.

“also if the labels are in curtain places” (Participant 2).

“Seams and labels, I find them quite difficult especially around my neck.” (Participant 6).

Others stressed the importance of the material that the labels, tags and seams were made out of.

“…if it’s got that stitching with glitter frame on it and also something woolen but almost have some glitter-flex in it, that’s quite abrasive” (Participant 2).

“it makes sense if this is polyester, because I have to cut all the tags out of my clothes because I hate the feeling of it…” (Participant 7).

Participants also reported that the pre-existing negative effects of the tags and labels became worse once they had tried to remove them from their clothes.

“if I cut them out it actually makes it worst” (Participant 8).

“…if you haven’t cut it out completely and the tiny bit that you left out will irritate you all day” (Participant 1).

#### Associations

This sub-theme presented how the effect (s) and understanding of certain fabrics were altered through associations with those fabrics through everyday living experiences as exemplified below. For example,

“that kind of rough material a bit between felt and sandpaper and it’s just…yeah…I can’t say I like it” (Participant 10).

“…it’s usually for sports stuff and it’s like it’s preparing you to do sports. It makes you feel proactive because it is designed to make you do active stuff like that.” (Participant 5).

Participants reported the effects of their childhood memories on their perception of certain fabrics.

“I have certain fabrics that have certain memories attached, like carpets at school which some of them were really uncomfortable” (Participant 2).

“This would make me little bit nostalgic in a way. Like childhood memories and it would make me feel safe because of that nostalgia.” (Participant 5).

“…when I had a bad day at school I would go to my room and pace and touch the wallpaper and that was because it was embossed, and it felt quite nice and therapeutic. Some fabrics that have slight emboss are quite nice…” (Participant 6).

### Theme 2: Body Sensations

The current predominant theme related to the magnitude of the physical body sensations that affected participants in their everyday lives. This theme entailed three sub-themes: Time-dependent; Parts of the body; Skin Sensations.

#### Time-dependent

Two participants stated that their level of coping with their tactile defensiveness was not predictable, since these were constantly changing as regards to the effect they would have on participants, as shown below:

“I feel that this is something that always keeps changing because some days I can handle like normal clothes, but then other days I just can’t find any clothes to wear.” (Participant 1).

“I suppose it’s a case of how I feel in the day and like, sometimes, I can cope with certain fabrics but other times it can be overwhelming.” (Participant 10).

The majority of participants reported that their tactile defensiveness was differently experienced when they were younger.

“… as I have become older, I am perhaps less sensitive.” (Participant 3).

“…probably it gets easier as you get older…” (Participant 7).

#### Parts of the body

Certain body parts were reported to be more sensitive and had more prominent and significant effects on the participants; most participants reported being more sensitive on the top half of their bodies instead of their bottom extremities.

“My shoulders, arms and sometimes my back are really sensitive but my legs I don’t have issues with that.” (Participant 6).

“I always hated things around my neck” (Participant 8).

Some participants also provided insightful explanations of the reasons why some parts of their bodies were more sensitive than others.

“there is a difference between touching something with your hands that you can easily get away from and wearing it all over your body because it’s like everything is more sensitive. But then again you get used to the stimuli in your hands but if there is a seam on your leg you are going to really feel something that you don’t usually feel.” (Participant 1).

“Especially near the neck, it’s quite sensitive because you obviously have just skin and bone and sensory dendrites there (laughs).” (Participant 2).

#### Skin Sensations

Participants expressed some of their most distressing and vivid experiences regarding fabrics. The present sub-theme encapsulated the magnitude, importance and impact that these unique tactile sensations had on the participants. For example, participants reported feeling needles and bugs on their skin when being in contact with an abrasive texture:

“I think the only way to describe it is shivers down my spine or like ants or stuff…” (Participant 9).

“All the little things that build up makes you a little paranoid that there is a bug on my skin or something.” (Participant 1).

“it is a bad experience when you feel like there are lots of little needles picking into you…” (Participant 2).

Participants also reported that they could feel tactile sensations even when they were not physically in contact with the specific texture(s) or fabric(s).

“…you can feel it even if your skin doesn’t touch the chair, you can feel it through your clothes…” (Participant 4).

“I’ve been at a party once that someone was wearing a velvet jacket and I couldn’t be in the same room as them.” (Participant 7).

“I am feeling uncomfortable and I am not even touching it yet…” (Participant 8).

Participants disclosed the side-effects of these negative tactile sensations; which encompassed some forms of self-injurious behaviors.

“…and then it makes you want to itch it and obviously it injures yourself because you want to keep scrubbing it and it’s quite hard.” (Participant 2).

“It can be itchy, and I start to scratch and sometimes I can cut myself and then you lose all focus and you can’t concentrate on what I am doing.” (Participant 6).

Others expressed feelings of distress and uncomfortableness when they were encountered with unwanted fabrics or textures.

“I get a little bit distressed only because it’s uncomfortable and it doesn’t make sense.” (Participant 5).

“my hands will physically sting, and it will make me highly anxious” (Participant 7).

### Theme 3: Coping Strategies

The final theme concerned coping strategies reported by all of the participants, and how the development of these was pivotal for their everyday lives, since these strategies enabled them to eliminate to some extent their negative tactile-related-symptoms, and encourage positive ones. The current dominant theme certain three sub-themes: Avoidance; Escape; To Feel Nice.

One participant gave a unique and clear perspective on how she/he perceived tactile defensiveness:

“If everything was cottony and bland and boring, I wouldn’t know what I don’t like and what I prefer. So, it’s almost having that balance of having that experience of fabrics and knowing ‘okay, well this is what I do and don’t like’…” (Participant 2).

#### Avoidance

This sub-theme related to how all participants expressed their strategies towards unwanted stimulation to prevent negative physical and psychological symptoms of tactile defensiveness. Some used strategies through their choices of fabrics and textures.

“…it’s easier when you are an adult because you can choose your own clothes” (Participant 10).

“I kind of have to pick my clothes quite wisely and even like furniture and stuff…Regarding my house, I chose it, so I already made sure that none of the furniture was made out of anything I didn’t like.” (Participant 7).

Participants discussed that they frequently used strategies when choosing their clothes, like for example touching and feeling the clothes first before buying them.

“I will spend a lot of shopping time touching materials until I find one that I like…” (Participant 9).

“… I’ve been known to rub the potential clothes that I will buy across my arm and particularly my arm and my neck which I am particularly more sensitive.” (Participant 6).

Some participants stated that they would avoid sitting on a furniture that was made from potential bad fabric(s).

“I can just avoid sitting or touching any other public fabric.” (Participant 1).

“…the other thing that affects me is where you choose to sit” (Participant 2).

The majority of the participants reported wearing something underneath their clothes in order to avoid getting touched by unwanted stimuli:

“…insisting on myself always wearing a t-shirt under a shirt because otherwise it is uncomfortable.” (Participant 4).

“I always wear long sleeves to cover all the parts of my body and therefore my clothing’s kind of gets in the way of any other public fabric.” (Participant 1).

In addition, a small number of participants reported using softener when washing their clothes in order to make them less abrasive:

“I use softener for my clothes a lot. So, I suppose it takes away that kind of feel from it.” (Participant 10).

“I wear jeans a lot, but I use fabric softener to make them a bit less rigid… Like if it’s not soft I’ll try making it soft and if I can’t make it soft then I don’t like it (laughs).” (Participant 9).

#### Escape

This sub-theme related to how the majority of participants reported using escape strategies in order to avoid pre-existing unwanted tactile stimulations.

“I just prefer to stop wearing clothes for a while or just stay in bed” (Participant 1).

“it’s just trying to remove myself from the fabric if possible” (Participant 10).

Other participants reported finding instantaneous ways to make themselves comfortable in an uncomfortable situation:

“…like that school carpet I had to make myself comfortable to be sure that nothings is going to stick into you if you got a pair of shorts…” (Participant 2).

“You would constantly be irritated and thinking of ways to adjust it and it wouldn’t make any sense.” (Participant 5).

The majority of the autistic participants stated that distraction was one of the most effective strategies to escape an unpredictably negative tactile stimulation:

“I cope with it when I put my mind into other things and distract myself. That’s the best way.” (Participant 6).

“I just think of something else and after a couple of minutes it’s okay.” (Participant 7).

#### To Feel Nice

Although negative experiences were more common throughout the data, the current sub-theme related to the strategies that participants were using in their daily lives in order to encourage positive tactile stimulations. In particular, some participants preferred comfortable clothes:

“It’s just a scarf but it could be quite comforting to wear so yeah, as I am walking around and then I stroke the material could be quite comforting and I generally like stuff like that.” (Participant 10).

“I really like wearing stuff that make me feel comfortable.” (Participant 5).

Participants reported being more able to cope with stress while having contact with the fabrics that they liked:

“I use the fabrics that I can cope with and that includes nature’s own fabrics and textures and I tend to use them a lot to calm down.” (Participant 6).

“a fabric can be comforting and say, with anxiety that can help a lot you know, just to calm you down and stuff” (Participant 10).

Some participants stated feeling secure and more relaxed when they were in contact with a good fabric (Appendix Table 3).

“You can rely on the fabric that it’s something that makes you feel, um…it is a strong word to say but, it makes you feel safe and at ease.” (Participant 5).

“… you feel more secure that you have something fluffier and softer” (Participant 9).

### Participants Experiences of Particular Fabrics

Participant’s recorded and transcribed narratives about the sampled fabrics were subjected to content analysis with data coded, categorized and presented in Appendix Table 3. The data revealed that participants preferred satin, denim and cotton, mostly due to the softness of these fabrics. Satin was particularly favored by 70% (n = 7) of participants. The majority of participants disliked hession, polyester, wool, and spandex, due to the abrasiveness of these fabrics which made them uncomfortable to wear, while hession and spandex were equally (90%; n = 9) selected as the worst fabrics out of the 7 samples.

## Discussion and Conclusion

The study examined a group of autistic adults’ experiences of living with tactile defensiveness and how this has impacted their everyday lives. These results were not made as an attempt to be generalized, since the study was exploratory, and could be used as a foundation for future research. IPA allowed the voices of people experiencing tactile defensiveness to be heard and for a deeper understanding to be presented. To our knowledge, this is the first study of its kind to do this.

IPA analysis showed that participants were very aware of fabrics and textures in their environments, and the clothes on their bodies impacted on their day to day functioning. Their familiarity with what they perceived as good and bad textures/fabrics enabled them to develop coping strategies to avoid and escape stressful experiences. Content analysis of the data revealed that participants preferred interacting with satin, denim and cotton, mostly favoring satin due its softness, comfortableness and light-touch feel. The fabrics that were reported to be the most difficult to cope with were: hession, polyester, wool, and spandex, especially, hession and spandex, due to their abrasiveness, and itchiness. It should be noted that just as each autistic individual has their own repertoire of symptoms, so each individuals in this study experienced fabrics in different ways. This means that our findings cannot necessarily be generalized to the whole autistic population. Nevertheless, overall, the data answered the three initial research questions, since (1) participants expressed how important fabrics and textures were to them; (2) they explained the effects that these had on their everyday lives; and (3) they reported the effects that the colors of the fabrics had on their perceptions.

### Awareness of Fabrics

All participants reported being aware (hyper-responsiveness) of the fabrics/textures in their surroundings, as well as the clothes that were physically touching their bodies. These sensations were expressed through feelings of irritation, discomfort or distraction/confusion towards restrictive, itchy and uncomfortable fabrics. Similar to previous studies (Grandin, 1992; Baranek et al., 1997; Jones et al., 2003) our findings suggest that autistic individuals are conscious of their surroundings due to hyper-responsiveness.

We also found contact with uncomfortable textures can cause autistic individuals increased anxiety (Markram & Markram, 2010; South & Rodgers, 2017) and distraction of concentration (Howe & Stagg, 2016). Similarly, some participants reported that particular fabrics used in schools have interfered with their learning and development, leading to negative associations and experiences of school. Indeed, unpleasant environmental textures may act as disincentives to the development of educational (Howe & Stagg, 2016) and social skills (Grandin, 2000; Markram & Markram, 2010).

Participants reported feeling hyper-responsive towards labels, tags and seams, which were perceived as uncomfortable, abrasive and irritating. Coping strategies included cutting-off the labels and tags from their clothes. Yet, the remaining material was still abrasive and impacted their stress levels and concentration. Previous studies supported similar findings; many autistic individuals avoiding tags on clothes (Kern et al., 2006) and wearing outfits with plastic seams (Ashburner et al., 2013). Whilst abrasiveness and restrictiveness of fabrics/textures have been reported as one of the main causes of irritation for participants, these fabrics are regularly found in public communal areas. Shopping bags made from hession-like fabric were particularly problematic for our sample; the finding was corroborated by Cascio et al. (2012) and Ashburner et al. (2013) who also found that this fabric was regarded as one of the most aversive textures for autistic individuals.

Conversely, our study sample demonstrated fabric favoritism. For example, muted colored fabrics were more favored compared to bright and pattern-based fabrics; these are more likely to be associated with visual sensory-perceptual irregularity, instead of tactile (Coulter, 2009). Furthermore, participants’ preferred soft and comfortable textures, like satin; previous research also reporting that autistic individuals enjoy touching soft and smooth textures (Cascio et al., 2012). Notably, participants’ showed sensory-soothing behaviors towards their favorite fabrics and reported being fascinated when they identified new soft fabric that they felt pleasing. Previous studies have similarly found evidence of autistic individuals constantly sitting and holding soft fabrics and textures (Ashburner et al., 2013; Dunn, 2007; Lane et al., 2011). Importantly, individual preferences have not been disregarded, since Ashburner et al. (2013) established that some autistic individuals enjoy touching woolen fabrics, and others dislike it. In the current study, 60% of participants disliked fabrics made from 100% wool, which might indicate either individual preferences or differences in the types of wool used amongst research studies.

### Body Sensations

Participants reported feeling a range of physical pain magnitudes towards unwanted textures and fabrics. Participants specified feeling needles pricking their skin and insects crawling on their bodies. These vivid descriptions may possibly demonstrate a lower pain threshold of autistic individuals compared to the general population (Cascio et al., 2008; Smith & Sharp, 2013). Participants reported hyper-responsiveness to tickle, itch and temperature; similar to previous research (Blakemore et al., 2006; Cascio et al., 2008, 2012). Some also described their worst sensory experiences as being in a vicious cycle that kept escalating their tactile sensory perceptions, and subsequently increased physical and psychological distress that often ended up in severe scratching and self-injury. Smith and Sharp (2013) found almost identical descriptions of their participants’ experiences with pain threshold. The qualitative approach in this study allowed the raw tactile feelings of autistic individuals to be heard (Smith et al., 2009).

Moreover, participants reported that their tactile defensiveness has changed since they were children. According to previous studies, autistic individuals do not become physically habituated to unpleasant stimuli, but they instead, produce coping strategies to become more tolerable towards undesirable textures (Ashburner et al., 2013; Smith & Sharp, 2013).

### Coping Strategies

Participants’ experiences with several stimuli appeared to help them understand how to better control their tactile defensiveness by using coping strategies. Similarly, Robertson and Simmons (2015) reported that their participants valued the knowledge of past negative tactile experiences and utilized coping strategies to gain control and subsequently decreased their anxiety. Participants reported avoiding unpleasant tactile stimulation; likewise, previous reports identified this as one of the main coping strategies used in this population (Ashburner et al., 2013; Smith & Sharp, 2013).

The participants in our study implemented avoidance strategies to escape overwhelming stimulations like removing all tactile stimulations from their bodies or distracting themselves from entering the vicious cycle. Distraction and avoidance behavior has previously been reported in other studies as coping strategies used by autistic individuals (Howe & Stagg, 2016; Smith & Sharp, 2013). These findings emphasized the importance of experience to learn strategies that enable autistic individuals to escape unpredictable and overwhelming tactile stimulations.

The tactile hyper-responsiveness commonly experienced by autistic individuals can enhance any textures perceived as pleasurable which can then be used as relaxation strategies (Cascio et al., 2008; Smith & Sharp, 2013). Choosing the right clothes and furniture or stroking/touching a specific pleasurable fabric were some of the main strategies reported. Repeatedly touching or stroking a specific fabric is a common strategy for some autistic people to experience soothing sensory experiences (Ashburner et al., 2013; Liss et al., 2006). Having coping strategies decreased stress and increased the subjective wellbeing of autistic individuals (Ashburner et al., 2013; Jones et al., 2003; Smith & Sharp, 2013).

The findings from the current study and previous research, have shown that contact with unpleasant stimulations could negatively impact the physical and psychological wellbeing of autistic individuals (Smith & Sharp, 2013). In contrast, these negative tactile experiences have enabled participants to discriminate what textures and fabrics they like and dislike, hereby allowed the development of more effective coping strategies for both avoidance/escape and the enhancement of pleasurable experiences.

### Limitations

While numerous ways were utilized to increase the reliability and validity of the study, limitations were unavoidable. Due to the exploratory design of the study, the participants’ current use of medication and previous experience of interventions related to sensory hyper/hypo sensitivity, were not investigated. These variables should be included in future research. Also, although gender differences were not under examination, there was an unequal gender balance which could have led to an unrepresentative sample; future research would benefit from an examination of gender differences. The small sample size of the study does not allow generalization of the findings. However, in accordance with IPA guidelines, the sample size was ideal for this analysis. Future research could focus on different analysis including larger sample sizes. Furthermore, everyday tactile experiences for autistic individuals are not as controlled and predictable as an interview setting, therefore, ecological validity was increased through the qualitative nature of the study.

Recruitment was carried out from one region/university, which could have compromised the generalizability of the results. The use of triangulation (semi-structured interviews; interaction with 7 samples of fabrics; and bringing their own favorite fabric), reduced the magnitude of the current limitation.

### Conclusion

The results of the current small scale study should be regarded as a stepping stone for future qualitative and quantitative research to examine the effects of fabrics on autistic individuals and to inform the autistic community, policy-makers, professionals, caregivers and the general community. In particular, we make the following research, practice and policy recommendations:

Research: We recommend a larger quantitative RCT study that uses clinical assessment tools for testing our initial findings of individual’s own experiences of different fabrics, as well as the soothing strategies they use.

Practice: we would recommend that environment such as schools, colleges and universities should consider using non-abrasive fabrics for communal furniture placed in study/work settings including accommodation blocks, libraries, and lecture theatres, in order to prevent distress and reduce non-community participation of autistic individuals.

Policy: policies such as the ‘Autism Good Practice Guidance’ (Department of Health, 2002) could progress their practices from considering the experiences of autistic individuals, in order to promote autism-friendly environments. It is important for our society to recognize tactile defensiveness in autistic individuals to better understand their behaviors and needs (Kern et al., 2006). By understanding tactile defensiveness, our community will increase autism-friendly approaches through the use of appropriate fabrics that could lead to social inclusion and better adaptation for autistic individuals.

### Supplementary Information

Below is the link to the electronic supplementary material.

Supplementary file1 (DOCX 71 kb)

## Figures and Tables

**Table 2:**
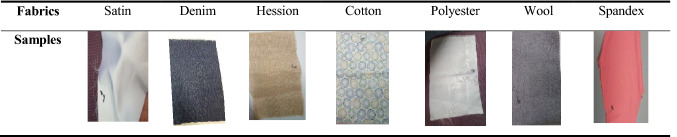
Seven Samples of Fabrics

The table shows the seven samples of fabrics that participants were asked to touch (if they wanted) and then explained how the encounter with these specific fabrics might have affected them

## Appendix 1

See Table 1.

**Table 1:** Interview questions

No	Interview questions
1	What does the type/texture of fabric mean to you? *Prompt:* is it important or not important to you?
2	Explain to me how these types/textures of fabrics affect your everyday life? *Prompt:* what areas of your life are affected most?
3	Does the color of the fabric influence the effect that certain fabrics have on you?
4	What is the right and wrong fabric for you to use? *Prompt:* What is that? Meaning, how does these affect you, if they do?
5	Are there any public places that you wished would use different fabric? And why?
6	**Giving Samples of Fabrics** Explain your feelings and thoughts as you touch each of the fabrics and explain how these might affect your everyday life
7	**Show their favorite fabric** Finally, please explain to me why you chose this fabric as your favorite one. How does this fabric affect or influence you?

The *indicated that participants interacted with either the sampled fabrics or their own favorite one(s)

## Appendix 2

See Table 2.

## Appendix 3

See Table 3.

**Table 3:** Content analysis

Fabrics	Percentage (numbers)	Liked/disliked	Reasons	Quotes
Satin	70% (7)	Liked	Soft, Comfortable, Light	Participant 6: “quite smooth and not irritating at all”
Denim	60% (6)	Liked	Soft, Light, Practical	Participant 9: “Denim is not too bad, I wear denim a lot”
Hession	90% (9)	Disliked	Rough, Abrasive, Itchy	Participant 5: “I wouldn’t wear it in a million years!”
Cotton	60% (6)	Liked	Soft, Neutral, Comfortable	Participant 2: “cotton is normally quite neutral, and I like that”
Polyester	70% (7)	Disliked	Abrasive, Odd, Uncomfortable	Participant 3: “It’s quite hard, and it feels very manufactured, and it doesn’t feel comfortable”
Wool	60% (6)	Disliked	Abrasive, Itchy, Uncomfortable	Participant 10: “this is awful”
Spandex	90% (9)	Disliked	Restrictive, Uncomfortable, Rubbery	Participant 8: “I don’t want to feel that uncomfortable”

The table illustrates the utilization of Content Analysis that was completed to explore, code and categorize the subjective experiences and interpretations that participants had for each of the seven samples of fabrics

## Appendix 4

See Fig. 1
