# Supplementary material for: Clothes, Sensory Experiences and Autism: Is Wearing the Right Fabric Important?
Source: J Autism Dev Disord. 2021 Jul 21;53(4):1495–508. doi: 10.1007/s10803-021-05140-3 (PMC10066095; doi:10.1007/s10803-021-05140-3)
Supplement: Supplementary file 1 — Supplementary file1 (DOCX 71 kb) [file 10803_2021_5140_MOESM1_ESM.docx]

**Clothes, sensory issues and people with Autism: is wearing the right fabric important?**

Chrysovalanto Kyriacou ^a*^, Forrester-Jones, Rachel ^b^, Triantafyllopoulou, Paraskevi ^a^

*^a^ Tizard Centre, School of Social Policy, Sociology and Social Research, University of Kent, Canterbury, Kent, United Kingdom, ^b^ Centre of Analysis of Social Policy, Department of Social and Policy Sciences University of Bath, United Kingdom*

*Chrysovalanto Kyriacou, [valanto_kyriakou1994@hotmail.com](mailto:valanto_kyriakou1994@hotmail.com) , 07763701661

Professor Rachel Forrester-Jones [r.v.e.forrester-jones@bath.ac.uk](mailto:r.v.e.forrester-jones@bath.ac.uk)

Dr Paraskevi Triantafyllopoulou [p.triantafyllopoulou@kent.ac.uk](mailto:p.triantafyllopoulou@kent.ac.uk)

**Interview 1:**

**What does the type or texture of fabric mean to you?**

Can you specify?

**Yeah, are fabrics and textures important to you or not?**

Textures are important, not emotionally because I suffer from…how do you call it…touch hypersensitivity? Touch defensiveness, something like that. Oh. Tactile defensiveness, that’s how they call it. They have so many names. So even when it’s not sort of, because you know how executive function sometimes, it could be one day and the other day you can’t. It’s a bit like that. It fluctuates how bad it can manifest. It sounds something really weird but…

**No, no, it does not sound weird to me**.

Tactile defensiveness or whatever, sometimes it gets really strong and everything is just bad. I mean, I have light sensitivity as well, but that is fine, because you can wear sunglasses or close your eyes, but when you are hypersensitive on your skin then you feel that there are bugs all over you like they try to come out of your skin and there is not a lot you can do about that unless you find a textile that you like and you will be okay all the time, but these are really hard to find a textile. I just prefer to stop wearing clothes for a while or just stay in bed or something unless I have to go out. It is important. I tried looking for seamless clothing but I none that could be comfortable.

**Explain to me how the types/textures of fabrics affect your everyday life? Like what areas of your life are mostly affected?**

I think, like any stimuli looks fairly negative. It’s sort of builds my stress levels throughout the day. It’s like constant nagging in your mind that you can’t ignore. Because I know if you have a constant sound all the time, your mind eventually ignores it, but this is just continuous, again and again and again and it just doesn’t go away. All the little things that build up makes you a little paranoid that there is a bug on my skin or something but it’s just the wind or seams or labels on clothing’s and it can make you incredibly distracted and lot more stressed. I mean, you sort of become used to being uncomfortable all the time, but you don’t overcome it in any way, you just become more tolerant. I think, sometimes it can become really frustrating and I would have a meltdown.

**Does the colour of the fabric influence the effect that certain fabrics have on you?**

They don’t change the actual feeling of the texture but I do like some colours over others. I mean if I have to wear it and it’s not a colour I like then I don’t really care. If it feels the same no matter the colour that’s fine.

**What is the right and wrong fabric for you to use?**

I feel that this is something that always keeps changing because some days I can handle like normal clothes, but then other days I just can’t find any clothes to wear. Also, there is a difference between touching something with your hands that you can easily get away from and wearing it all over your body because it’s like everything is more sensitive. But then again you get used to the stimuli in your hands but if there is a seam on your leg you are going to really feel something that you don’t usually feel. I think the smoothness. I really like smooth, not rough textures and also very soft textures. I mean, if I had to wear something too soft it would be tickly which I wouldn’t like to wear, but mainly without seams and labels. It would be fine if they didn’t have a lot of seams and labels

**How does the right and wrong fabric affect you?**

When you are hypersensitive, I don’t know for others but for me the smallest touches that feel the biggest. So when a fabric is rough, it’s not really rough, but it has all these small different tiny little problems on it and you can feel them the most and that makes it most frustrating. Which is why the labels, if you cut the off and if you haven’t cut it out completely and the tiny bit that you left out will irritate you all day.

**Are there are any public places that you wished would use different fabric? And why is that?**

I guess sometimes the library. Like the chairs are kind of itchy. The texture they use particularly is uncomfortable, I mean I wouldn’t lie down on them. But there are other things, like sensory stuff at the library that are more important like the lights and that there are lots of people. I mean if you are one of those people that don’t cover up your body, you would be uncomfortable, but like me, I always wear long sleeves to cover all the parts of my body and therefore my clothing’s kinds of gets in the way of any other public fabric. I mean if that wasn’t the case and usually this is very rare for me, the one thing that would bother me would be the seats sort of a problem. Although I guess there it is not an issue if you have really smooth, leather seats. I guess it would benefit if the library changed their seats, but it’s not the most prominent problem. My most important issues are with the clothing I am wearing because I can just avoid sitting or touching any other public fabric.

**Now, I would like you to touch each of the fabrics in front of you, and if you can, please tell me your thoughts and feelings about them and how they might affect you in your everyday life?**

**Satin:** this is really smooth on the one side, but on the other is a little bit rough, I don’t like the other side. This is not bad, it’s nice. This is very smooth.

**Denim:** I think jeans are generally soft and they are not that bad to wear. I think one of the issues sometimes is just really specific and you can’t determine why this is a better fabric than the other one. Yeah, this is normal clothing.

**Hession:** this is rough. I don’t think that anyone would ever use this, I wouldn’t be able to wear that. But like the big circular chairs in the library are using this kind of fabric.

**Cotton:** cotton is nice I think and as you can see the edges are not rough at all, so I like it a lot. It’s not too soft but it’s okay. It’s a good texture but it’s not my favourite one.

**Polyester:** it’s like the shower curtain (laughs). Polyester is cool but I think a big issue would be like seams from this because it would make them really obvious. The labels are just there to make you feel bad. I mean, if I touched labels by themselves it would be fine but in the way they are set up on the clothes is not fine.

**Wool:** no I don’t like this one. No.

**Spandex:** this is nice. It’s like the sports clothes. I hate the clothing that puts a lot of pressure on me, like tight clothing, but I have always liked the texture of spandex and it’s an issue because then it stretches all over you. But it’s really good because it’s soft and seamless sort of, and especially if you turn it inside out is very seamless. I like the texture but I don’t think I would wear it because of the tightness.

**So, did you bring your favourite fabric?**

The skin of some people is my ‘favourite fabric’ in the sense of touching it etc. but obviously I couldn’t bring someone here to show you. My favourite fabric to wear is the fake leather. There are two types though, one is nice and feels more leather. The inside of this trousers is good as well as the outside because both sides are soft. When I bought it I couldn’t stop touching my legs which made me look really weird, but it’s so smooth and soft that it doesn’t tickle at all. Especially because it’s old it makes it even softer. The inside is nice but there are seams and I cannot wear these often because of the seams, which is a shame because I like it a lot. I tried to wear it twice but when I am really sensitive I can’t. I need to try again though to see if anything changed or maybe find ways to cover the seams. The only way to use it is to wear it for a few minutes to feel it and then take it off before they annoy me, because of the seams. They make me feel happy, most textures make me feel happy. My flatmate has really soft skin so we cuddle, she doesn’t mind of course, or I can just touch her arms or hug her for long time. It’s really fascinating when you find something soft and you just need to keep stroking it. You know, sometimes you get super soft fabrics in stores but they are too soft to wear them for long periods of time. Generally, most of my feelings about fabrics are annoying but I really enjoy touching the fabrics that I like, or to stroke them for long time. You sort of content, but not necessarily excited.

**Interview 2:**

**What does the type and texture of fabric mean to you?**

It is important, I have curtain fabrics that have curtain memories attached, like carpets at school which some of them were really uncomfortable so you would be able to be sitting somewhere or be able to wear something that is not very abrasive on your skin because I find some fabrics that almost feel like sandpaper and make you feel like they are rubbing your skin off so it is really important in terms of clothes and also sitting down when you are wearing shorts that you are not wearing something that is going to rub against your skin and literally it feels like it is scraping your skin off, which is quite unpleasant. I think that those standard school carpets in Great Britain. They have standard school carpets and it is a cross between Hession and wool and it is wobbly and it’s probably navy blue or green and it’s in the corner and you all sit on it and it doesn’t get cleaned often so it’s just disgusting but it certainly is also how the environment not hopefully had something like a fabric on the wall or the door if you touched that what he effect would be as well. I mean we don’t have many fabrics on doors at home but carpets I do. Also you would wear socks in the house, like cosy socks that are not touching the carpet or don’t go from carpet to laminate because that would feel odd, going from hot-to-cold as well because the laminate is much more cooler, so it’s also picking up on temperature as well.

**Explain to me how the types or textures of fabrics affect you everyday life? Like what areas of your life are mostly affected?**

It certainly affects what I choose to buy and where and also if the labels are in curtain places and what the labels are made out of because if the label is quite cottony or quite satiny it’s fine, but if it’s something that’s quite coarse or if it’s got that stitching with glitter frame on it and also something woollen but almost have some glitter-flex in it, that’s quite abrasive. And I think the other thing that affects me is where you choose to sit and where you choose to position yourself in the room and as well as obviously light and noise, having something like a different fabric may impact if you sit on a chair or something. This chair is absolutely fine but some of them where is really plastic makes you very uncomfortable or if they are like that school carpet I had to make myself comfortable to be sure that nothings is going to stick into you if you got a pair of shorts or if for example something is really heavy or very light. I mean, I have a quite heavy blanket on my bed because I always sleep with a duvet because I pretty much like the weight and certainly having that heavy blanket is quite good, it is quite soothing. Certainly in terms of wearing fabrics I prefer things that are not as heavy so something like cotton, like a jacket which is quite useful and I like things that are quite stretchy as well so not really really tight jeans, you obviously got jeggings and kind of denim leggings which are quite stretchy and actually denim which is quite stretchy but if you can’t get that proper lean feel of it you can’t wear it. I couldn’t tolerate that when I was much younger. I also think that certainly before 15 or 20 years ago there were less variety of fabrics. Certainly over here there wasn’t that kind of stretchy denim that there is now and there was more synthetic clothing so I had more high-waist and high-neck t-shits as well which I find sometimes could be a bit pressing on the neck so certainly I think there is better range now than they use to be. But who knows what fashion would change, but has got much better. A bad experience with a fabric would be, like the hession, and certainly the little fibres feel like they are almost digging into you. This is a specific example, but it is a bad experience when you feel like there are lots of little needles picking into you and then even if it’s gone it feels like it still feels like it’s itching as if you’ve been picked with a needle but then it still hasn’t gone away so it’s quite unpleasant. And then it makes you want to itch it and obviously it injures yourself because you want to keep scrubbing it and it’s quite hard. In terms of a good experience with fabrics, it’s an interesting one, I could go for a good experience that does not cause any abrasiveness, like the cotton. That’s quite nice, getting into fresh sheets is always very nice but then there is also cuddling our rabbits which is obviously which isn’t a fabric is linked to the cushion I have brought. Very soft, and I have the association that rabbits are not as stupid as many humans are (laugh). But they are very soft, it’s almost that with animals that unconditional love and they are also very very soft which isn’t fabric related. Things like purr because one of our rabbit purrs so certainly the vibration of the purr and the fabric or kind of their coat which I guess its linked to the fabric I brought is very very relaxing. So it’s the fabric and or feel of the rabbit along with the purr, so when you touch something that feels like the rabbit you then have that association. In terms of a negative emotional feeling, the effects of it depends on how long it goes on, because touching the hession like now does nothing to me, but if I had to wear hession all day I would get quite stressed because I wouldn’t know, it would feel like I couldn’t move and I would keep moving to keep the pricks out of my skin and then I’d gradually get more and more agitated and the more agitated I get the more stressed I get and the more I move the less clearly you think because it goes round and round in circles. And certainly in terms of feeling and emotions, is stress, elevated anxiety and never wearing hession ever ever again (laughs).

**Does the colour of the fabric influence the effect that certain fabrics have on you?**

As I said before, I can’t wear anything that is navy blue because of the association I have with my secondary school when I had to wear blue blazer, blue jumper, blue trousers and it was just like ‘ugh’ (disgusted). Um…it’s quite odd. But then again they had their walls painted red which is much weirder. And like a brown stripped blouse and you are just like ugh (disgusted)… (Laughing). So I don’t normally wear navy blue because it reminds me of school and I definitely don’t buy black shoes or anything like that because it reminds me of school. I can wear denim that’s blue, that’s not a problem but do prefer a not navy blue denim because again it reminds me of school I don’t wear attractive patterns like this (ironically talking about the cotton sample), but I do prefer things that are a bit more muted and so obvious, but I think that’s more taste than the actual association with the fabric as such. I think the other thing in terms of fabrics is associations with things like carpet and wall coverings because churches have a very standard carpet and schools have very standard carpet when you sit down almost it has that associative feeling when you sit on someone’s house and they have the same colour carpet. Like standard church carpet and chairs which would probably be red chairs and red carpet which in schools would be blue or green carpets so it is certainly the association that ‘is it going to feel the same and things like that?’ The colour of the fabric would not necessarily change the physical effect that the fabric has on me, probably not. I mean, I have in here (bag) my cushion that I brought which basically reminds me of the rabbit we used to have and I like the feel of it generally but I like it even more because it is exactly the same colour as my rabbit and it has the colour of the tammy and the colour of the outside and it feels just like her so it can make it better but I never had an experience of making it worse.

**What is the right and wrong fabric for you to use?**

Not this delightful hession (laughs ironically). I wouldn’t wear that and I don’t know anyone that could wear that to be honest. Certainly in terms of wool in which I have had problems in the past, the sample wool is okay, but it’s like when it’s knitted and it has loads of those glitter-flex in it I find glitter abrasive since it’s kind of plastic-y. Especially near the neck, it’s quite sensitive because you obviously have just skin and bone and sensory dendrites there (laughs). I think certainly it depends what you mean by the kind of usage. I don’t like anything that makes me sweat so I wouldn’t have polyester on chair or the bedding because even though it might seem very luxurious like satin as well, it would be quite odd as well and certainly the kind of silk-y, satin-y and polyester-y things although they feel nice I can feel the fact that it doesn’t allow my skin to breathe so something like cotton is not very polyester-y. I couldn’t deal with silk and all that kind of stuff. It’s just not. It looks nice but also some silks are very polyester-y sometimes and it can feel rubbery but very stiff. Whereas something like cotton doesn’t feel stiff and I think it lets your skin breathe so um… I think certainly there is that evidence for my skin that knows when it can breathes and when is can’t. So in terms of clothes and also bedding and chairs is really important.

**Are there any public places that you wished would use different fabric? And why?**

Schools should not use that horrible carpet (laughs). This is about 23 years ago so I think they don’t still have that carpet per se. I think that now everyone has their table and they just have a carpet that is all over the school instead of having a specific sitting carpet area which I think I a bit better. I think certainly more cottons for things like, but I don’t know why they sell so many beddings with satin and silk and polyester, just because it looks nice and it is shiny it doesn’t have any merit to it, really. And I think also as far as chairs goes, obviously they have to make it durable but it would be nice if there were things like throws and covers were available in rooms that you could almost put on top. Or for the summer that is really hot would be nice because it can rub a bit but just picky. But it’s quite nice to have a mixture of fabrics. If everything was cottony and bland and boring I wouldn’t know what I don’t like and what I prefer. So it’s almost having that balance of having that experience of fabrics and knowing ‘okay, well this is what I do and don’t like this and this is why I do and don’t like it’ rather than everything being the same and not knowing why you like something or not, so it’s having that balance.

**I would like you to touch each of the fabrics in front of you and if you can, please tell me your thoughts and feelings about them and how they might affect your everyday life?**

**Satin:** I will try not to break it (laughs). Um…I will have a feel of the wool first. I will actually start with hession which is the one I don’t like. The satin is a little bit odd because it’s silky on the outside and rough on the inside and when you rub it you can hear that noise which annoys me and I don’t like it at all. But again nobody likes that side. It is stretchy which is good, but the underside is quite odd I think. But certainly something like that is very nice to feel but then when you get hot you can feel the sweat and that’s what I generally find with satin. So it’s almost like it’s too good to be true.

**Hession:** I would never wear it and I don’t know who would ever wear it. It reminds me of those things you get in um… kind of like a bag or something. I think that’s the only practical use for it. Maybe chairs. It would certainly be nicer if it was slightly less abrasive like less little things popping out, but it’s a very practical thing that I wouldn’t wear. That would make my skin itch.

**Wool:** they wool hasn’t made my skin itch which is nice. Almost reminds me of a felt like fuzzy felts like as a child, it reminds me of that. This wool is much nicer than the wools you buy in shops that they have loads of little things sticking out which I find quite difficult, but this is a nice wool.

**Spandex:** this is swimming costume stuff. It is not abrasive, it’s quite nice actually. It’s not something I’d wear. I think also in terms of faults feelings and it reminds me of going swimming. So everything is going to have their own memory. It reminds me of going swimming and I think we used to have swimming lessons at primary school and I used to hate them and I think I used to go swimming the same day later and I could already swim and the teacher we had was like Miss Trunchbull out of the Matilda (laugh). I don’t know how she got into teaching to be honest after all she did. She was actually a primary school teacher and she used to chuck things around the classroom and loads of other stuff. So it actually reminds me of swimming lessons and having her shouting at me.

**Polyester:** which reminds me of neck curtain and it feels a bit drying on my skin. It is not something I would wear but it certainly something you put against your window to stop people peering at you. It reminds me of putting neck curtains up on my nuns house and we live in my nuns house now and we’ve got windows about 4 meters wide and we had fun to put the poles and then that stuff (laughs ironically).

**Cotton:** which is probable one of the better feeling ones but the most oddly patterned ones (laughs). It almost looks like it is a cell. I think it reminds me very much of the fabric I have in my bag, which is why I brought a pillowcase I have at home which is quite cotton-y. So it’s quite neutral and I think that’s what I quite like. Whereas some of them are overly nice or overly abrasive, the cotton is normally quite neutral and I like that.

**Denim:** this quite a nice denim because it is a light denim. I wouldn’t wear a really heavy denim. But this is quite a nice denim. I don’t really have any faults on the denim other than the fact that that this particular denim is quite neutral but sometimes I find denim quite heavy and so but this is nice because it’s quite light.

**Now we will talk a little bit about the fabric that you brought with you if that’s okay. I would like you to explain why you chose this fabric and how does this fabric affect your everyday life?**

Yes. Okay. I find it hard with the word favourite fabric so what I did I picked 2 fabrics. One that is pleasant and that I know is pleasant across the board and one fabric that feels like someone that I used to have at home, our rabbit and I actually bought the cushion because it looked like and felt like her. Certainly in term of fabric, it feels like her because… (pause)…I don’t know, it think I bought it because she was my rabbit but also I bought it because her fur was very different from our other rabbits because she was a brown rabbit but she was also a very muscly-big with big brown hair so her fur was very different to our white rabbits and our brown rabbit and black rabbit. They all got very different fur but she always had kind of thick soft hair especially underneath her chin and on her tammy which is almost like that middle and it’s basically a lighter brown. It was there when I felt like I need to stroke it, but also because it’s from Primark and its fine (laughs). But also some cushions often are quite decorative whereas this one is decorative but it has a function in terms that it is very soft and it does look nice and it adds in the aesthetic of my bedroom and reminds me of my rabbit so it has everything (laughs). Whereas the bedding is just something I picked up this morning and again it is from Primark but it doesn’t feel very cheap. Certainly I enjoy having fresh sheets and if it wasn’t so much hard work to change my sheets every day, I can’t do it while doing my masters (laughs). I chose this beddings because it’s not abrasive and it’s very neutral and they are supposed to be relaxing so again there is an association there. Also, even when the sheets are washed I can still smell the washing detergent so it’s very fresh smelling. So it doesn’t feel abrasive and it smells nice but not too strong which is almost like fabric again coming in with other bits but fabric is being the cake and the smell is the cherry on the top because if it smelled nice but the fabric was rubbish I wouldn’t bring it.

**Interview 3:**

**What does the type and texture of fabric mean to you? Is it important? Is it not important?**

You know what? It’s not massively important to me but it is to a degree. Um…it was more important when I was younger and I used to have more issues, but now I am a lot sort of tolerant. Yeah, I like textures like sort of feelings when you get a new note-pad or something, like just feeling the paper or something like that. But, in terms of sensory issues and I know this is not what you are studying but I am quite sensitive to noise like I’ve got to be in the silent area, but textures is not something that massively affects me to be honest. So yeah, it’s not that important to me.

**Explain to how the types/textures of fabrics affect your everyday life?**

I think the main thing in my everyday life is wearing socks. Um…so that’s been an issue for me when I was younger and it is still is an issue to me now to a degree. Um…in that I like particular, I am kind fuzzy you know when they’ve got that hem on the top because it feels a little suffocating so I’ve got to have socks that are really soft and comfy and not too much fabric when it gets too worm, not too little. Um…and it takes me quite a while to find a set of socks that I like and I buy a lot of them and it generally just makes me feel quite uncomfortable. And then also like t-shirts and things I can only feel the hem, like where they sew it. Yeah, I really notice that and it feels like it’s the wrong point and there are a few t-shirts that I’ve got which I wear if I am running out of other clothes where I don’t like the fabric so I try not to wear them. In terms of how it affects me, it gets a little bit frustrating. Its more frustrating when I am trying to find something that fits because like, I’ve got socks that fit where I don’t have an issue on a day to day but it’s when I try to go out and buy those socks or if I get socks which aren’t as comfy as I thought it’s just frustrating. I mean, I can wear them you know, it’s not going to cause me a big-big issue but it’s just uncomfortable.

**Does the colour of the fabric influence the effect that certain fabrics have on you?**

I don’t think so, no. I am trying to find more to say but I just can’t find a different way to say it. So, no it doesn’t affect me in any way.

**What is the right and wrong fabric for you to use?**

I like things that are soft and sort of quite fluffy and I don’t like things that feel plastic-y. Like this one (polyester) that feels uncomfortable whereas something that just feels a bit more genuine, neutral.

**How does the right and wrong fabric affect you?**

No, it wouldn’t necessarily affect me. It would just be uncomfortable.

**Are there are any public places that you wished would use different fabric? And why is that?**

No, I have never noticed that to be honest.

**So are there no public places that would make you feel okay or uncomfortable?**

No, no to be honest.

**Now, I would like you to touch each of the fabrics in front of you, and if you can, please tell me your thoughts and feelings about them and how they might affect your everyday life?**

Satin: I don’t really like it because it’s quite um… it’s not soft, it feels quite false. I know they are probably awful so it sounds like a stupid comment, but it just feels very manufactured. It’s almost a bit slippery slightly but not soft. Yeah.

**Denim:** I don’t normally wear jeans and I have never really wore jeans and I don’t know why to be honest because I don’t actually find them uncomfortable but when I wear them I’m fine but I always kind of have in my mind that jeans are uncomfortable and that I don’t like them.

**Why is that?**

I don’t know, I think there are better fabrics that I prefer, so maybe it’s just that or maybe is also that as I have become older I am perhaps less sensitive. But I can find comfortable jeans. It’s also quite hard feel, you know, it’s not fluffy like wool, it’s quite rough.

**Hession:** I actually really like that. Its texture is like massaging almost when you touch it with your hands. Yeah, that’s quite nice. But I guess it’s like a shopping bag or something. I wouldn’t wear it, and I don’t know if anyone would to (laughs). I just really enjoy touching it but not wearing it.

**Cotton:** again, it’s soft and it’s a bit like softer and fluffier that satin. But it’s also, it’s not soft-soft. It still feels like hard and manufactured feel to it. On the inside it’s nicer I think. It feels a little bit polished on the outside, I don’t know if it’s been sprayed or something. I don’t dislike it a lot but I don’t particularly like it.

**Polyester:** Yeah, I definitely dislike this one. It’s quite hard and it feels very manufactured and it doesn’t feel comfortable. It’s not warm as well. Say like this one (wool) it feels a lot friendlier to my skin. So I used to have lots of cuddly toys and some of them had this (polyester) kind of material on them and it used to really bug me when I was little. I didn’t like cuddly toys with this material on them. You can smuggle it but it feels uncomfortable, but if the cuddly toy was made up of wool it would me a lot softer. So yeah, I don’t really like this one.

**Wool:** yeah, I do like this. It’s just soft and it’s not only smooth. It feels comfortable again I don’t think it’s a wearable material to be honest.

**It is wool.**

Oh! Then yeah, I would wear it in that case. It is a bit calming and relaxing.

**Spandex:** again, it’s like satin or polyester. It’s got that false kind of feel. It’s not soft, it’s hard. It’s also something that wouldn’t be relaxed, I think that it would cling on you. It wouldn’t affect me so much to be honest, apart from hession and polyester. If I had to wear any of these, it wouldn’t do that much to me or I wouldn’t have much of a reaction to it. Generally, it is what I feel about these fabrics but at the same time I don’t feel that strongly about them. Like for a swimsuit, it would be okay to wear it if I had to. Talking for me and textures in an ideal world is not a priority, whereas perhaps other people might feel like they face more challenges with textures.

**So did you bring your favourite fabric? Can you please tell me something about it?**

So I decided to bring a sock just because socks are the things that I find that I do have more issues with and bother me so I think it’s almost like a kids socks with the pictures and that (laughs). I chose this one because it’s not too soft, it’s not too light, not too heavy. Like when I wear it I don’t feel it. Whereas with a lot of socks I will sometimes find that I am perhaps feeling the sock. With this one forget that it’s on which I guess it’s quite nice you know, it’s not itchy at all, it’s not scratching me, it doesn’t feel heavy, it doesn’t feel light. It’s just there. I mentioned the hem of the sock earlier and I am referring at the part on the very top, which this one doesn’t have and the fact that it gribs a little bit it doesn’t bother me and its soft isn’t it? You know, I have got some socks that are a lot harder but this one is just soft and comfortable.

**Interview 4:**

**What does the type or texture of fabric mean to you?**

Some are comfy and others are not and I sound incredibly basic, but I am not sure what you are trying to get out of my answer. Because other fabrics are comfy and others are not, others look nice and others don’t. I don’t know if there is anything else to say

**What I mean is, if fabrics are important to you or not?**

Not particularly, no. Obviously if it was like something very obviously uncomfortable then yes, but obviously if it was something particularly comfortable then that would obviously be really really nice. But I don’t think it is more important for me than for any other people. But I am not really sure, I haven’t thought about it much. .

**I would like you to explain to me how the types and textures of fabrics affect your everyday life? If they affect your everyday life of course.**

Um…the only real example I could think of is um…insisting on myself always wearing a t-shirt under a shirt because otherwise it is uncomfortable. I mean, obviously if it’s hot I will just wear a shirt just without a t-shirt but I prefer not because I like having a t-shirt underneath. It doesn’t bother me that much. I will have two woolly jumpers that I would wear, but obviously I wear stuff underneath it and I am always drawn to wear a collared shirt so the actual fabric doesn’t touch my skin so that’s not a bother. It happens sometimes when I wear a scarf that I really like but it can get sometimes annoying, a bit irritating because it touches my skin. If it didn’t wear a t-shirt underneath my clothes I would probably be a little bit annoyingly aware of the fabric and probably have a contemplating and maybe awkward just wearing a t-shirt underneath but I would worry if it’s too hot, but then usually after that, obviously I am doing other stuff as well, and during the day you kind of adjust into it. Like jeans, shirts, it’s only really a bother on the immediate aftermath of me having to put it on. But once I start doing stuff during the day, it doesn’t bother me. Um…also, this is not particularly relevant to me but I am sure a lot of people get this, but if it’s summer and you fall asleep on leather sofa then yeah, that’s not great because you stick to it. So that is not ideal, because I need to do a lot of catching up during the day because I wake up significantly earlier than the average student but I also end up getting distracted on the internet, like every student, late into the night, so it’s like burning a cantle on both its ends and also because um…all sorts of things. I just fall asleep everywhere. Yeah…I need a lot of sleep. Which is very unfortunate because I am an English Literature student, when I do reading, and we do a lot of reading, I generally fall asleep. So it doesn’t particularly help the situation (Laughs). That’s why now I have to do it sitting up or have to sit at a hard chair at the desk to stop that happening.

**Does the colour of the fabric influence the effect that certain fabrics have on you?**

I am not actually too bothered about the colours and textures of the sort of fabrics you generally encounter daily and certainly not around the university because they use very agreeable to the size and touch of the fabrics they use.

**What is the right and wrong fabric for you to use?**

It’s like you can’t really know what you think about that. Because generally we all wear fabrics that you will wear or fabrics that you kind of buy them because they are comfortable. You wouldn’t buy them if they weren’t comfortable in the first place. Obviously the clothes that you are wearing generally they are going to be comfortable anyway. But also you will think “oh, I want to wear the white t-shirt today instead of the red one”. The point is that you like all of them, that’s why you have them isn’t it? But in the process of actually buying clothes I suppose if it’s says 100% cotton and wool or whatever, then obviously that is going to be a plus just because it’s more comfy and I don’t know if everyone would do that, or is an autistic thing or not. Um…I wouldn’t buy something made out of polyester but I think that’s probably more to just that it’s cheap and tacky anyway. Um…I suppose another example would be, when I am choosing a pullover I would either choose this one (pointing at his/her woollen jumper) or choose another one I have which is like very comfy because it’s not actually on your skin but you kind of want to wear it and it’s the placebo effect of knowing that it’s comfy.

**Are there any public places that you wished would use different fabric? And why is that?**

Again, I haven’t experienced anything like that before and I am trying to say something along those lines. But I really don’t know because who ever really encounters much fabrics in their life anyway. What kind of places were you thinking?

**By public places I mean places that the majority of the people encountered at least once in their life. Like the school, university dorms, lecture theatres, hotels, houses, the library etc.**

I used to read at the library but I don’t do that anymore but for other reasons, but one minor annoyance, that it doesn’t bother me that much but it’s just mildly irritating was some of the round-backed chairs that is like the arm and the back are all linked up together. Those ones are found in the numerous occasions where I would fall asleep deliberately or otherwise on them, it’s like the fabric it’s self is obviously uncomfortable to sleep on, but obviously it’s a chair not a bed and I suppose you could say you don’t want all the chairs to be too comfortable otherwise that would make everyone sleep and it won’t function as a library but an Bed and Breakfast (BnB) (Laughs). It would be just kind of nicer if it was more comfortable especially given that it is meant to be comfortable, then why would they have that and not like a dining table or something. But when you are reading it can be quite nice to have a comfortable chair to read. I don’t know what specifically to suggest but it would fit some kind of fabric that was more pleasant to the touch because you can feel it even if your skin doesn’t touch the chair, you can feel it through your clothes and you feel the texture of it. But again, it’s a very mild example. I know the kind of thing about the proper like the ‘rain man’-‘Sheldon Cooper’ stereotypical Asperger. But I don’t have that, and I know that some people would not tolerate that and it would get on their nerves because of the hypersensitivity, but it’s not so much the case with me.

**Now, I would like you to touch each of the fabrics in front of you, and if you can, please tell me your thoughts and feelings about them and how they might affect your everyday life?**

**Satin:** it’s thin and weird, but I couldn’t imagine this feeling warm except if you are wearing a cardigan or something, but this one is very thin for that. It’s quite strong and there is not flexibility. It’s very heavy and very light at the same time, yeah, I don’t like this one.

**Denim:** Actually I am wearing denim right now. You wouldn’t be able to do a chair out of it but it’s obviously jeans. It’s better because it’s practical above anything else, and it is quite comfortable as well.

**Hession:** Definitely not, whatever this bird-sack is. No. This is scratchy, horrible material. I would never wear that.

**Cotton:** It’s definitely different from the satin and nicer. I would wear this because my t-shirts are made out of this and because it is the most used fabric. I like it because it’s not itchy, it doesn’t rub or anything and it all kind of flows very neatly with the human body. And now I am over-analysing why t-shirts are popular (laughs). Yeah, it’s really comfortable in short.

**Polyester:** Is that polyester? Because I always wondered what polyester actually mean to be. So, definitely not in a million years! it has the same problems with the satin but x100 worse. You know when someone has a reedy voice. This for me is like a reedy fabric. It is very thin and light but also harsh to the skin. This would irritate me 100%.

**Wool:** I’d wear that. That particular type of wool specifically yes, because it feels warm somehow and it feels like it’s generating its own heat. That’s why they still make stuff out of wool, like scarfs and things. It is often times itchy but at the same time the material compresses and flexes about in such a way that its very nice. That’s what generates it being very warm on other things.

**Spandex:** oh this is swimsuit material. So, no in a million years, but I think that’s just the association of the material. But if I would just concentrate on the material alone, I wouldn’t wear that as a t-shirt. I have a running t-shirt back at the house but it’s not the same. But when you wear swimwear they usually have the netting inside that it complicates the feeling because you are not just judging it on the merit of the material but yeah…in general I find swimwear really uncomfortable even if they are just dry it’s like the material has an annoying way of brushing lightly against the skin. Yeah…very annoying slight touch. Obviously anything that comes in contact with water and that’s in contact with you is always very uncomfortable, some more than others, but that’s the general principle. The texture itself has that annoying touch of quality that is just annoying.

**So now, can you please tell me something about the fabric that you brought today?**

Yeah, it’s the t-shirt I am wearing underneath my jumper. (Participant shows the t-shirt to the interviewer: white cotton t-shirt). I haven’t thought before this point what actually is my favourite fabric. I decided on this t-shirt because this kind of cotton that is particularly made for t-shirts is very comfortable and I thought what else could I have chosen that is comfortable and I wear it, and I couldn’t find something else better than my cotton t-shirts. Maybe like my pyjamas but I think this one is more towards the classification of my favourite one. For me choosing my clothes is obviously does it fit right or look nice, those are the two primary concerns for me, but if it then happened to be particularly uncomfortable for whatever reason then obviously it wouldn’t matter if it’s nice or if it fits. I care more about the quality of the clothes I am wearing, and if they look nice that’s a plus. Being comfy means it comes with a territory for that.

**Interview 5:**

**What does the type or texture of fabric mean to you? Is it important to you? Is it not?**

It helps for personal comfort, like physically and mentally. I mean is not like a big thing but I really think that it does help in making you feel reassured in some way. Well it depends if its clothes or a material on a door or a chair or something and it depends on the context. I think it’s useful to consider the reassurance and I think it’s good if it makes a person feel at ease. Um…because it irritates you if it’s a bad fabric. If it’s a good fabric it is satisfying, but if it’s a bad fabric and it makes you feel uncomfortable, you notice it immediately. So I would say that it is important to consider that. But it depends if its clothes or something else. So yeah, I think it is important to me, because if it is fabric I don’t like, then I’ll notice it, but if it’s a fabric I like, I might not notice it, unless it’s a fabric I don’t like, maybe, I suppose (laughs). Like when I am looking at this (pointing at Hession) and I am thinking, if I had a shirt like this, I would be really really irritated, Yiak, (laughs).

**Can you explain to me how the types and textures of fabrics affect your everyday life? If they do?**

I suppose it is similar to what I said already. I suppose they do because if it’s a fabric, you know, whether its clothing or whether it’s a surface of something that I have to touch, or sit on or if it’s a fabric that I come into contact with a lot, then I will remember it and so it helps if it’s something comfortable. Because as I said, if it’s a fabric that irritates me I will probably want to avoid it, so I suppose that’s important as well. So if it’s a bad fabric that I will need to interact often with then I will probably avoid it because I will prefer a good fabric. If it’s a good fabric then it will make me a bit more relaxed and I’ll remember that it is a good fabric because it makes me feel more relaxed. The bad effect that I am talking about its like it sort of irritates you because it doesn’t feel nice, or because it reminds you of something else that irritates you in another way, then it won’t me a good experience and a good memory, but when it’s a good one, you will feel reassured and that its comfortable and you can trust I suppose. You can rely on the fabric that it’s something that it makes you feel, um…it is a strong word to say but, it makes you feel safe and at ease. Well, that’s the important part if you interact often with that fabric. Yeah.

**Does the colour of the fabric influence the effect that certain fabrics have on you?**

Yes, sometimes. Again, it’s more likely that it will affect me because, again this is a really Asperger’s thing to say but it’s more likely to affect me if it’s a colour I don’t like, because if it’s a colour I like I will probably appreciate it but then not realise why I appreciate it, until I realise that it’s because it’s not a colour that I don’t like. The other thing, that’s even more Asperger’s, sometimes I will be a little bit annoyed if the colour of the fabric doesn’t go very well with its surroundings. I don’t usually make a big thing about it, or get triggered by it but sometimes I will get a little bit annoyed, just personally, if a fabric colour doesn’t make any sense with the things around it or the room. Yeah, sometimes it will annoy me, but I will only really get annoyed when it is a colour I don’t like. When it’s a colour I like, I am more likely to remember it, but its not a big thing. I generally look at the colours and notice them. If it’s a really unique colour then that would interest me because I am more likely to remember it. So if it’s a colour I don’t normally see in many places then it will interest me. I wouldn’t say I have a special relationship with memory, but I would say that my memory specifically, I don’t know whether its because of my Aspergers or its just because of me, maybe it’s a combination of both, but I was lucky I knew about the condition since I was seven years old, anyway that’s not the point. The point is that I think my short term memory its not really any better than or any worse than anyone else, I think, but my long term memory is kind of unusually good because like, I remember, I am not saying I remember everything from my childhood but sometimes I remember completely random details from really long time ago that it doesn’t really connect to anything else, it’s just ‘Ah!’ (Laughs). Also, because of that, I think it is important to value memory and to appreciate it so I think in this context it’s sort of um…depending on the type of fabric, I would say, if it’s a unique colour I am more likely to remember it because its interesting and unique and like I said, I’ll probably remember a colour that doesn’t go with the surroundings and it will annoy me a little bit, and when it’s a colour I like on a fabric that come into contact with often then I would remember it more. It depends on the specifics.

**What is the right and wrong fabric for you to use?**

Again, that depends if it’s something I wear, or something I sit on or something I touch. Um…I think, um…I haven’t really thought about it before. I think, that there are two things in fabrics that would make me automatically not like it. So one thing that I don’t like, and I think no one likes it is if the fabric is extremely uncomfortable to wear, in another context maybe it’s not that uncomfortable, but by looking at this (Hession) it looks like its abrasive to the skin. The second thing that is again quite Asperger’s is if the fabric doesn’t make any sense, what I mean by this is that if the fabric look like it’s made in a counterproductive way, like the pattern or the stitching or something. If the fabric is made and it doesn’t have a specific purpose or if it’s not suited to its purpose. That would irritate me. Sometimes I don’t like when something is made of a material that doesn’t make sense with its specific purpose. There isn’t any wrong fabric since all this is subjective, but if a fabric is really uncomfortable or it doesn’t make sense in terms of where it is placed, there I will get a little bit annoyed. Um…it triggers my OCD, but I don’t like using OCD because that’s a different disease and I don’t have the full version of that, actually maybe I do (laughs), I just have Asperger’s, which as you know, I have tendencies which are similar to OCD but less severe. So yeah, it can occasionally trigger my OCD tendencies, but I am less peaky about these things now, but sometimes it can be influenced by that and it just means it if it’s something really bad or if it doesn’t make sense, I will just look at it and think why did no-one think to change that and why would someone think that is a good idea and it makes me worry a little bit about peoples (laughs), okay okay, that san extreme example because I don’t usually think that far ahead unless I am really depressed on that day (laughs). But if there is definitely a non-sensible context that I personally think it’s really stupid that might make me think why would someone think of a stupid ideal like that and it would make me worry a little bit I wouldn’t say it triggers anxiety or something, but like, just a little bit of annoyance and sometimes little bit of confusion. If I would say I was uncomfortable like this (Hession), it is really useful for a potato sack, potatoes are really under-rated, I mean I was born in Russia (laughs). I mean, this material (Hession) is really uncomfortable, the only problem with that is that I know it is uncomfortable if I wear it so I wouldn’t wear it, so it doesn’t trigger anything. Sometimes the thought of having it on you might make you a bit uncomfortable but nothing major. The right fabric for me would be something that doesn’t make me feel uncomfortable and it’s contextually making sense.

**Are there any public places that you wished would use a different fabric? And why?**

There are a couple of lecture theatres, I don’t remember exactly the rooms that the fabric doesn’t make any sense and so I notice when something doesn’t make sense because I think ‘oh they should have used this instead to make it softer there, or make it more fit for purpose here.’ And again, it doesn’t trigger anything because I can just avoid it, but sometimes I do realise that when I am sitting in a place I visit often, I think of how better it would be to change the material used for this particular purpose. With transport also, like seats and on trains and planes, every now and again, you think that there is a fabric they provide whether its for, I don’t know, drying your hands or the door or whatever, sometimes I notice that its kind of, they could have used a much more logical fabric for that. So yeah, I do notice, but it’s more on the back of my head rather than on the front. Its only occasionally that I notice something and it irritates me. Sometimes when I am in a workplace, you know, a place where I have to be there for a long time or a study place sometimes, but if you are at work you have to be there, so sometimes if I am in a place like that if they use a fabric that is uncomfortable then I will get a little bit distress. For example when I worked at a hotel, sometimes a cloth that they provided I would think that they could have used something better instead of insisting in using a rougher one, or something I have to sit on, or change the bedding, occasionally there I would think that they could have used a different fabric for that. So yeah, I get a little bit distressed only because its uncomfortable and it doesn’t make sense, so if it’s the two together and also I have to work with it for a long time then it would get quite irritating and maybe a little bit distress, only because of the combination. I am not completely distress, you know, I am not going to get PTSD or complain to my boss saying ‘oh, you should be using another fabric for that’ (laughs). So yeah, if it’s the two things combined for a long time then they are more likely to remember.

**Okay, now I would like you to touch each of the fabrics in front of you, and if you can, please tell me your thoughts and feelings about them and how they might affect you in your everyday life?**

**Satin:** oh this is like for sheets and stuff, like silk but not quite. Um…I think of this, since I have worked in a hotel. This fabric is used for specific purposes. I don’t know, it doesn’t make me feel any kind of bad emotions. I wouldn’t wear it as a shirt, am not sure It would serve the purpose of a shirt even though I’ve seen people wearing it. It wouldn’t be really terrible to be fair but again, its more suited for sheets or duvet covers or something. Its not completely uncomfortable, its reasonably strong for what it is and it looks good quality and it looks respectable I suppose, and comfortable and safe. Again, it should fit a purpose, it would make me feel fairly comfortable and safe, maybe not to wear but as a fabric because its useful.

**Denim:** This is jeans, and its relatively strong and obviously popular material, and it is strong for what it is, therefore it would make me feel safe and because it’s so recognised as a material, it would also make me feel safe and transparent, in that respect. But also, it’s not very soft and so it wouldn’t make me too comfortable. It would sometimes make me feel a little bit under pressure because it is the type of thing you wear when you have something to do, and it would make me feel that I have something to do rather being in my pyjamas all day (laughs). It’s a versatile material which is great but it’s really not good when it’s raining and it would make me worry a little bit if I was to wear it.

**Hession:** God damn, I wouldn’t wear that! (Laughs). I would not wear that as a shirt but it’s a very very useful material for its specific purposes. It is strong and probably cheap to make and people have been using this material for many years. It also reminds me of Russia because they had lots of sacks full of potatoes and tomatoes, which is very stereotypical but you know its true (laughs). This would make me little bit nostalgic in a way. Like childhood memories and it would make me feel safe because of that nostalgia. It’s not a bad material, although oh my god, I wouldn’t wear it in a million years (laughs)!

**Cotton:** this is used in everything, it would make me feel fine I think. We wear it all the time. Um…I don’t think it would cause any particular emotion. It’s certainly comfortable with it. It’s not hard like that (Hession) but it’s not soft like that (satin), or like all the others. It’s kind of in-between? So it’s quite versatile and neutral and recognised. Now that I don’t need to show off so much (laughs), I prefer clothes like this that are comfortable instead of wearing something uncomfortable. I now like to wear clothes that make me feel more relaxed and not making me feel under pressure or something. For example, I have a motorbike leather jacket that I had since I was like 15, but it’s really solid and until I wore it a lot I felt like I was wearing an armour or something and it was really hard. Even sitting in a train is painful. The rule is not to wear it unless there is a festival or something. Whereas nowadays unless there is a specific event were I want to show off or whatever I really like wearing stuff that make me feel comfortable. Only because I get paranoid about things very easily so definitely nowadays I try and think in terms of what makes me feel relaxed and able to just concentrate more on what I am doing. I think now this is more important for me rather than when I was younger, now that I am thinking of it more. Not like consciously but it makes more sense.

**Polyester:** shower curtain (laughs). This is a bit like that. It is interesting because it’s kind of again serves some kind of purpose but it’s also thin and it keeps water out usually so in that respect its useful and protective and again to wear it, it depends why you are wearing it, like sports and stuff. I supposed in some ways it’s the opposite of that (denim) because it would make me less worried about stuff like rain and things. In that respect it’s more reassuring. Yeah, it’s not extremely soft but it makes up for being useful. I would wear it for like sports stuff and certain purposes because I would feel safer.

**Wool:** This is pretty cool. This is definitely something that brings childhood memories, you know, in Russia everything is freezing all the time. I guess this is the comfortable version of Hession in terms of the nostalgia. This has something with that when they forced me to wear wool and I didn’t realise why it was useful at the time. It’s a bit of nostalgia. It’s also warm and comfortable but it’s not ideal for the rain but it’s not bad. But if you have a jacket made of that, it’s like it’s not designed for it. It’s thick enough that it keeps the rain out so it could be worn in many situations. Not in summer. But it’s very kind of safe and warm and protective and it also has that memory of wearing it to protect yourself. There is also the risk of getting too hot but (laughs) but that’s not a big issue. The only thing that I would say, is that if I had to wear it with nothing underneath it would feel itchy and stuff. It would be irritating but I don’t know who would wear it without anything underneath to be honest. I have a coat that reaches the floor and it’s made out of wool and Kashmir, yeah, it was quite expensive but like it’s really warm and it looks amazing and I had it for a long time now so yeah. It’s amazing, especially if it’s a little bit windy (laughs).

**Spandex:** This is the swimming stuff. Um…well this is interesting. It’s kind of malleable and light and different from the others. It’s usually comfortable (laughs) it’s pretty good material for swimming and sports. It makes you feel like ready, because it’s usually for sports stuff and it’s like its preparing you to do sports. It makes you feel proactive because it is designed to make you do active stuff like that. That is something good about it. Um…it’s versatile because it’s good for the rain and it dries very quickly which is brilliant (laughs). The only thing is that it’s kind of fit for active purposes it’s maybe not the most versatile thing. So again, you wouldn’t wear this everyday just for the sake of it (laughs). Well you can but because it’s made for a purpose it’s kind of like ‘oh I should be doing something active’ and in that respect I contradicted myself their but you know what I mean (laughs). Yeah, it’s all just different purposes and memories and different feelings they give you. So, yeah, I would wear that but only for swimming, not all the time.

**So, now I would like you to tell me a few words on why you chose this as your favourite fabric.**

I couldn’t think of my ‘favourite’ fabric, but I bought something that has sentimental value to me and has fabrics that I like. There are like three fabrics on this but mainly cotton linin. It’s a shirt that dad sent me a few years ago and he liked it so much because it was such a specific style. It’s not something special. He liked it so much he didn’t want to throw it away, so he sent it to me and maybe after five years later, now I am big enough so that I can wear it. Well…this (pointing at a part of the shirt) which is not the material that I am referring to. This is sort of like linin which is quite good because it’s sort of good when you are in a hot country because it’s thin and breathable and also fairly strong, which is quite good because I used to live in Spain, but that’s not the material I was talking about. The material that I am talking about is quite interesting and I don’t really know what it is, it looks like linin or cotton maybe and a little bit of silk or something and I actually think that it’s these materials combined. It’s like malleable, it’s like rigid and I don’t have to iron it often. I don’t know if I want to talk about this or the linin part. The whole shirt has sentimental value to me because dad sent it to me because he was like “oh, I had this shirt and I just want to give it to you” and I found it quite nice because he thinks I am the best option. Because I lived in Spain I think the combination of these materials is under-rated because if you are in a hot country, I hate wearing shorts, I personally think they don’t suit me at-all, if you have something that is made out of these materials it serves the purpose of keeping you cooler but you can still covering yourself up and it’s easy to maintain, it doesn’t need to be ironed very much and it dries quickly. It is also comfortable because none of those materials are sort of like closed. Most of these (7 interview materials) are quite closed. So for example if you are in the desert these materials (favourite shirt) are breathable enough that you know you are not going to sweat like crazy, but if you wore that (wool) you would be insane (laughs). The thing that I like about this material is that I don’t need to worry about sweating too much or being too tight, something like this makes you not worry about your body temperature reacting the wrong way. I think that’s quite good because again, it’s reassuring and it’s also a material that is so light, and kind of soft and loose, and you don’t really feel that it’s on you. So like when you are wearing jeans, even though they are comfortable you know they are on you. If I am wearing linin trousers it’s better than wearing shorts because they are kind of lighter and bigger, so I feel like they are not really there. So it’s good when you feel that something is not there because it means it’s comfortable you know. You are not irritated. If you wore that (Hession) you would feel it all the time especially near the chest. You would constantly be irritated and thinking of ways to adjust it and it wouldn’t make any sense. So I suppose that I bought this for sentimental reasons but also like the fact that you don’t have to worry too much about the possibility of being irritated by it and you don’t have to worry about temperature control and you know I like it because it’s easy maintenance and I think is all those factors that make me feel reassured and it makes me worry less and it doesn’t trigger any particular irritation, and it’s also nice in touch. I was thinking of bringing also a pair of trousers that I’ve got that are kind of velvet that I really like, but I was thinking that I cannot wear them in the summer because it’s too hot and I think that’s why I bought this thing instead even though it’s a bit confusing because it has two different materials, but I think the common factors of those materials is sort of the point that I was trying to make. Like you don’t feel that it’s there and it’s just easy to keep and you don’t have to worry too much about temperature control. I mean this is a bit terrible in the winter but you can just wear more things. Yeah, I think that’s the main theme that I thought when you asked me to bring my favourite material and I feel comfortable with. So yeah, I don’t really know what my favourite material in the world is with those things in common and feeling reassured because of those reasons.

**Interview 6**

**What does the type or texture of fabric mean to you? Is it important to you? Is it not?**

It’s not one of my initial sensory issues that I really think about other sensory issues actually cause me a little pain whereas the sense of touch normally just leaves me uncomfortable and also there is always choices. There is more choice on whether something, and things like that. So for me the sense of touch and fabrics is more on my terms if you know what I mean. Like you can’t obviously determine the sunlight and things like that so I find it more immediate to cope with. I have sensory difficulties in regards to fabrics but and even textures which are natural. However it’s easier to put strategies to deal with them, but you know, it can make things difficult some time. For me it was when it was school, I used to hate the fabric of the school uniform so you know, sitting in a fabric that you don’t like, you can’t concentrate. You lose focus. You potentially lose most of your learning because of that. Obviously when I was a lot younger, Autism wasn’t such a big thing and you had to wear it whether you liked it or not. For me personally, fabrics affected me more when I was at school. Thankfully I haven’t worked in a job where I had to wear a certain uniform, so I got away with that because it would essentially affect me if I ever had to wear a uniform or certain fabrics that feel awful. Um…I hate clothes shopping, even like fashion (laughs). Um…I have to go to the shops. I cannot order online because that’s how I prefer to do a lot of my shopping. Online. But with clothes I don’t shop online, because otherwise I’ll be sending half of the stuff back. So, it’s just not worth it, so I have to go to the shops myself. So I suppose in a way it’s getting me out of the house and do things that other people sort of like doing but yeah, that’s the one thing it makes me do which I don’t like and that’s because you can’t touch things online. It makes clothes shopping just not worth it you know. So even when you buy cotton, sometimes it’s not the right cotton. You know, it could be different types of cotton and I don’t know. Um…even two same t-shirt or similar ones, but for some reasons each cotton seems to feel different and um…again I have to feel every bit. I’ve been known to rub the potential clothes that I will buy across my arm and particularly my arm and my neck which I am particularly more sensitive. It might look totally bizarre when I am doing it, but if it doesn’t feel comfortable in the shop then it’s not going to feel better later. So I have to make sure that the whole thing feels comfortable which can look rather amusing to some people, or very strange but I’ve got to do it just to feel comfortable. I also tend to stick to the same clothes which could be boring to some people. I don’t like certain fabrics like track-suits or shell. And it’s not the way they look that makes me uncomfortable, it’s how they feel. I tend to wear jeans because they are comfortable. So, but even then there are some jeans that just don’t feel nice. I have to feel things before I can acknowledge and assess that it’s going to be okay and I have to be near the whole thing. It doesn’t matter if, um…let’s say there was a beady dress, event tough it’s on the outside because my arms will be touching the beads I couldn’t handle that at all so even when I sort of buy dresses which is very rare they have to be very sort of plain. It’s a shame and it’s quite gutting really but just to be able to cope with the situation obviously that I am getting into with the piece of clothing I am wearing I have to be comfortable because if it’s not, it makes what I am doing really difficult. So you put the two together, being in a situation which I don’t want to be in but then not feeling comfortable with my clothes. The two put together, the anxiety levels are going rocket (upwards gesture). So yeah, it can have quite an effect. Regarding the library, some of the fabrics on the seating can actually make it quite uncomfortable so yeah. The fabric I think at Uni needs to be thought about you know, even if it’s just the sits.

**Can you explain to me how the types and textures of fabrics affect your everyday life? If they do?**

Um. I have already done that so I just skipped ahead. It can dictate but then, there are more choices compared to my other sensory issues like noise and lights. But then I can’t just block everything that’s going on in the world and how the world works and I cannot block out the sunlight so, i can’t turn the world over (laughs). So yeah, I have more choice in what fabrics I wear but sometimes when I am hot that fabric can change. I like buggy clothes and not tight. Obviously when you get wormer things start feeling a little tight so it can really aggravate you. Also with shoes, like when you are warm your feet expand and it again can feel a little sweaty or something, that can be irritating. So, I think subconsciously I think about it. I do look at the weather a lot to determine what I should and shouldn’t wear. Um, yeah. I think certain things as well. I don’t like sleepers as well, but I think that’s because of other things really. But it could also restrict clothing that could potentially be comfortable. So you don’t know really. It’s just being aware and to constantly think about it. Like my brain doesn’t switch off. It’s always on the go. Even at night. So that would be the kind of thing I would think after. Like, ‘what’s the whether going to be tomorrow’ or ‘oh, I haven’t seen the weather, I better go and check’, you know, because that would determine what clothes I am going to wear the next day and you know, the amount of layers. It really does have quite an impact if it keeps you awake I suppose. Which makes me wonder you know, ‘what type of clothes I should wear?’ because I am used to wear casual clothes and sometimes I need to dress up a little bit more because I have something that’s happening and I don’t quite feel comfortable in fabrics. That’s because it makes me anxious. I mean the clothes get irritating and I don’t know whether it’s my anxiety that causes the irritation or because the clothes are on me or that’s the irritation itself if that makes sense. I don’t know, so yeah. It sounds plausible. So that can have an impact I think.

**Does the colour of the fabric influence the effect that certain fabrics have on you?**

Yes. Um, that’s shocking. That peachy colour, I can’t stand it anyway (talking about the spandex sample). I would struggle to wear clothes with colours that I didn’t like. There are certain shocking colours, like really bright ones, patterns. Most of my clothes are really blunt in colour and quite plain. I have few items that are patterned but are more of geometric because I like making patterns out of them, but random patterns make me struggle a little bit more. Although this one (cotton sample), the colours are actually nice and the pattern is actually calming, but no. I prefer the more plain. Oh, stripes. Oh my God. They can actually make you feel quite dizzy depending on the thickness. Summer dresses oh…sometimes I just have to look away from people and I might seem quite rude but I am not it’s just the summer dress they have on are too fuzzy and have far too much bright colours that I cannot cope with that. I struggle going shopping during the summer actually because are brighter. I prefer autumn and winter colours. So yeah, bright colours are not nice. Oh! The luminous colours. The 80’s where a nightmare for me. Everyone was wearing those everywhere (laughs). Like those colours that are so bright and they glow in the dark, oh…awful…absolutely awful. Yeah. Shiny fabrics I struggle with the glare. Yeah.

**What is the right and wrong fabric for you to use?**

Seams and labels I find them quite difficult especially around my neck. That is a major issue for me. Some are worst that others. Some companies are worse than others. Like seams and some clothes shops I probably wouldn’t even go in because I know the seams are really harsh and they use a thread that I wouldn’t say it hurts… but irritates really-really-really badly so tend not to go to those shops. So, it’s how the material is made that makes a huge difference on the fabric as well. What is made of, where I am wearing it on my body, my legs are not that sensitive as my top part. Hats are okay. I actually like wearing hats. I don’t know if it’s because of my mind-set, but I don’t like feeling cold because I get cold very easily so to me a hat keeps me warm so that kind of feeling is better almost than other hats possibly feeling. I tend to wear loads of hats on before I buy them to feel comfortable and most of the ones I wear have a fleece on the front so again I am kind of choosing what to wear. For me it’s more about how it feels than how it looks so obviously for most people it’s the other way around whereas I couldn’t give a damn on how it looks. It’s how it feels that is more important for me so that’s where I might differ from others. Things have to feel comfortable. Seams are the things that I particularly have to check for. Um…so yeah. Wool is difficult for me. Silk I find difficult sometimes. I find it rough actually because you can feel the lines in it and I can feel where the lines are in silk and it’s just irritating. Some silks are worse than others. Silk is not good for me suppose that is more expensive but, no. in regards to even shopping actually there seems to be marketing things for all these little textures and things like that and that can impact on whether I buy things as well you know. There are things that I have seen that they are doing it with this (Hession) rather than a plastic bag. I wouldn’t even think to buy that it wouldn’t matter unless I could get someone to carry it to my car and then someone to my house because I would really struggle to hold it. There are even some plastic bags that I struggle with. There is some that are more recycled but the feel of them but, don’t get me wrong, I’d rather use recyclable bags but the way they feel they almost feel like rubber and I sometimes struggle with rubber. Picking up anything that’s from rubber can be really difficult as well and these bags that I would prefer to use because of the environment I actually struggle to hold because of the way they feel. If they had something where the handles are, that would be helpful purely because I would prefer to use them. Again, you are restricted in what types of bags you buy maybe back pucks. I mean my one is a study one but I wouldn’t wear that on my skin so I always wear clothes but if I had to because I was too hot that would really annoy me. Just the feel of it is just irritating. There are some materials that are embossed that I actually kind of like. I mean how extreme you want to get here. Like in regards to wallpaper they can be irritative, but they can actually benefit me. For example when I was a child my mom placed the most horrific floral wallpaper and she couldn’t understand why I couldn’t settle and then she asked me to pick and I picked one that looked like the old 70’s kitchen tiles because they were geometric and it was in my bedroom. There were three reasons I liked it. One was because I could make patterns out of it and my mom always knew that when I had a bad day at school I would go to my room and pace and touch the wallpaper and that was because it was embossed and it felt quite nice and therapeutic. Some fabrics that have slight emboss are quite nice. It was also my favourite colour-blue and white and I used to do many shapes and patterns and maths equasions. You could be out and you would lean on something and it would feel so horrible that you wouldn’t want to touch it again. And these wallpapers could be both denim or fabric type and they are so itchy and it’s quite annoying. It can have an effect. My friends tell me to stand over there and I say ‘but I am looking at it and it even looks itchy’ (laughs). Oh, and plastic leaves could be really annoying when you accidentally touch them and I prefer nature. I am quite happy with real plants because plastic ones have little spikes which can hurt on touch and feel like they are stabbing you. Also, marketing materials that they use can feel horrible so I don’t tend to buy them because my children won’t go out shopping so it was just me. It’s amazing how fabrics you know, it’s going to make me more aware now (laughs). No, no, no. I have many other things to be fixated now. But there are other fabrics that feel lovely though like the fleece. I also love leafs. Like their feel because they are slightly embossed and there is something in the leafs that I like about them when they are in autumn and they are slightly wet when they are on the ground. So there are a lot of textures in the environment that could also help to decrease stress. Sometimes walking on grass in bare feet can be nice. You know, instead of walking on the path, you can walk on the grass sort of thing. You know, it’s softer under your feet. It’s just coming up with strategies that help you to counteract the stress. But you can’t always get I right, like when I buy a shirt it feels nice and then it doesn’t because obviously a different temperature of my body reacts to the fabric so yeah, I don’t always get I right. Even now. I use the fabrics that I can cope with and that includes nature’s own fabrics and textures and I tend to use them a lot to calm down and just stay clear of number 3 and six (laughs). It’s about building up strategies. People that do marketing and people that do interior design need to have an awareness of not just people on the spectrum but people that have sensory processing disorders and even people with ADHD and a lot of developmental disabilities have sensory processing. It impacts a huge amount of people and I think the more awareness is not a bad thing.

**Are there any public places that you wished would use a different fabric? And why?**

Um…buses. I went on the bus earlier and it was actually quite of itchy. Stage coach bus and it was quite an itchy fabric. Um…so that can be quite of difficult, so I have to make sure I don’t touch the seats because when I get agitated can take over and get fixated. Especially around my neck. For example, I have a polo neck which I would normally feel comfortable in and I am used to it or worn it several times but say if I am a bit anxious where I am or it’s a bit too hot It can be itchy and I start to scratch and sometimes I can cut myself and then you lose all focus and you can’t concentrate on what I am doing. For example I remember I was at an autism function and it was really cold outside and obviously I didn’t think in advance that day and I was literally sitting there and it started being really itchy and by that time I couldn’t focus, I couldn’t tell you what happened during that time, and everyone was talking and by that time I have cut myself because it was that extreme and I had my friends telling me ‘just go, it’s fine, you are cutting yourself now, just go’ they knew I haven’t taken it in, they told me what happened the next time I saw them (laughs), so no good. Things like seats where the back of the seat… so if someone is wearing a vest top and it’s an itchy fabric that might be irritant. Um…what else. Cinemas are okay. It’s mainly when you go out to restaurants and things like that, um…some fabrics are definitely better than others. My children are all on the spectrum and we are pretty much the same so we kind of look (laughs), you know, kind of the same wave length. Um, I think my partner struggles a bit (laughs) by not being on the spectrum even though sometimes I think he is but (laughs), no he is not. But he is very aware of our sensory needs because we’ve been together for a very long time and I think sometimes he prepares sometimes more than we do. It obviously has an effect on us. There have been times where we just stared at each other and we both asked to be moved to another table and sit somewhere else. You know, we haven’t said why I should need to. There has been a couple of times that I have asked to be seated elsewhere and they are always kind. There are also things like work that they don’t give a choice to people but to wear uniforms without considering if they have any sensory difficulties and I know many children that are struggling now because their uniforms have been changed, and those where my issues when I was at school. School and work uniforms are issues for me if I had to wear one. I think that’s about it. If I think of anything else I’ll let you know.

**Okay, now I would like you to touch each of the fabrics in front of you, and if you can, please tell me your thoughts and feelings about them and how they might affect you in your everyday life?**

**Satin:** the underside of this is rough. The upside I could wear it. If I had to wear the other side it would be quite irritating. So yeah, you can tell it’s quite irritating and impulse I would struggle. Maybe not so much the smoother side but I wouldn’t get anything because you would worry if the other side could touch you and that would become an irritant. But if both sides were smooth it would be fine. That’s actually not too bad, quite smooth and not irritating at all. My wedding dress had to be doubled up actually because the other side caused me annoyance, so I had both sides satin. The smooth part. Yeah, that was nice, she was fantastic. That was many years ago. It would need to be doubled up if I had to wear that.

**Denim:** I could wear this on my legs because my legs are not so sensitive, but I couldn’t wear it on my arms. Would you mind if I? (Touch the fabric on neck)

**Of course, yes.**

**Denim:** oh. On my leg is fine, but I couldn’t wear that as a shirt or anything like that. That’s very much for both sides as well. Yeah. My shoulders, arms and sometimes my back are really sensitive but my legs I don’t have issues with that. Otherwise I would have been all painful, no, no.

**Hession:** Oh! No, no. that is horrible! Yeah, no that’s not nice. That’s horrible, it’s like little spikes. I couldn’t do a job or wear these. No, not good. Very-very hard, no (laughs).

**Cotton:** the pattern is not shocking as such and I actually find the colours quite of soothing but yeah, um…I find circles to be easier to look at. Even though I like geometric patterns, they have to be really geometric like triangles and squares so I can make patterns out of them if I can. If the fabric is not making patterns out of it, the circles are easier to look at. Don’t ask me why. They just are (laughs). But yeah, that’s fine.

**Polyester:** yeah, polyester (laughs). Seams are also very difficult. Yeah…not comfortable. I mean, I am okay with linin, more that they are more natural fibres, I don’t know but no, that doesn’t work because pure wool is just shocking. So itchy. No that doesn’t really work. Not as bad as some, but I don’t wear polyester. I can’t wear it for until it gets really annoying.

**Wool:** that’s itchy isn’t it? I won’t put it on my arm because I know I will start scratching. It’s also the after effect. Like, even if you take it off it can still be on your mind for a little while so if you took it off you would know it’s still there you know. You’ve scratched it, so it might take a while to kind of zone back down. I cope with it when I put my mind into other things and distract myself. That’s the best way. Or sometimes you just deal with it. Sometimes it doesn’t look nice because I am literally scratching and scratching but eventually it will just go. You know. But, my friends try to distract me when I am really-really scratching. I remember once when we went out and I wore a top that I thought would be okay because I do get it wrong, and it got irritant and the night went on and I had to go to the toilet with my friend and change tops so just that I can get through the evening. So yeah, my friends have been quite good (laughs). They understand because their children are on the spectrum they get it almost. So yeah, number six is not a good one for me. It’s really-really itchy. So again, I have to be very careful with bed linin. I really do have to be really careful with bed linin. My children seem to like them, even though they are on the spectrum, I can’t cope with them at all. So it’s literally 100% cotton and again, I still have to be careful. I couldn’t sleep with just a dovet on because they so itchy so again is things like that could potentially be restrictive. Certain sleeping bags, I have to be really careful with patches in sleeping bags and I have to literally get in and you know rub it with my arms (laughs) because you know, I just got to see what it feels like which that could be a little bit annoying so yeah.

**Spandex:** again. Smooth side and rough side. The inner side would irritate me. Again very much similar to the first one. Yeah, I would struggle. I think pretty much for the same reasons as that one although this isn’t as bad as number one. I don’t tend to go swimming to be fair, because I am not a strong swimmer but yeah, I find swimming suits quite tight obviously so I don’t feel too comfortable, so I don’t tend to go swimming as such. To some extent the fabric affect that because it’s just doesn’t feel comfortable. I mean, if they were like the outside part on both sides it would be fine but that fabric would get on my nerves. It’s not as bad as number one, the inside because that is harder. Yeah.

**So, now I would like you to tell me a few words on why you chose this as your favourite fabric.**

It’s basically the super soft evening gowns. It’s kind of like a fleece and it’s just very soft and there is nothing that could irritate me because it’s so soft. Obviously it has its main purpose of holding your dressing gown tight but I do use it for other things. Um…I tend to wear a lot of hoodies with this material which I have got one today. And I use this material to keep my anxiety levels low. If I know I am doing something that I am apprehensive about I would just sit and touch it and stroke it. That keeps my anxiety down. I always take a little bit of it especially if it’s somewhere where I have to be focused. Like a lecture or supervisions. So, I just wear my hoodie and I just stroke it and it keeps my anxiety levels down you know and I manage for some reason to keep more focused and it helps with my concentration though I think it’s important to me to be able to do that. I used to do it when I was at school and I was consistently told off to stop because I was annoying and of course again I wouldn’t get much learning done because I wasn’t coping. And obviously behaviour occurred. Not so much now (laughs) this was when I was at school. At school I had challenging behaviour and also the Autism wasn’t picked up so I was just you know…I had sensory issues and didn’t like change and everything was constantly changing while not been told of anything changing so you know…there was a lot going on. So if I had a piece of material which is a strategy I use to this day you know, it would have made a huge difference. So I think its important for me to have something I like. Children tend to use more like fiddle toys but for me it’s a bit of fabric and my God, it would be odd to sit there and fiddle with a toy (laughs). So I think I find things very difficult if I was told to stop. For me, it just keeps me grounded. Keeps me able to focus and concentrate and yeah. I think without it you wouldn’t get as much from me.

**Interview 7:**

**What does the type and texture of fabrics mean to you? Are fabrics important to you or not?**

Yeah, they are relatively important because there are certain fabrics which I can’t even be around or even just looking at them, for example velvet. I can’t. It just makes me cringe and I kind of have to pick my clothes quite wisely and even like furniture and stuff. Like when I was looking for my new uni house, I had to make sure that none of the fabrics I chose was velvet, so yeah, it is quite important.

**Do these fabrics make you feel anything in particular you mean?**

I think like, the materials I don’t like is bad for me because it kind of makes me…I don’t know how to explain it. I get different feelings with different materials. But when it’s bad, it kind of makes my skin crawl so yeah, it is important for me.

**Explain to me how the types and textures of fabrics affect your everyday life?**

Um…well. I am used to describe the clothes that I know I like so all my clothes are quite similar in the sense that I like baggy tops and stuff. But I can’t wear tops that are too tight and the same like shoes and socks and I only have to wear them only when I go out, and as soon as I go back home, I take them straight away off because I don’t know…I just feel quite trapped. So yeah.

**What areas of your life are affected most?**

Like if my friends are wearing velvet I can’t be around them but they all know not to wear it around me (laughs). For example last Halloween my friend was wearing a bunny costume that was made out of velvet so I couldn’t go near her for the whole night and she like, understood that you know (laughs). So yeah, I tell my friends not to wear that around me because it’s the material I have the worst issue with. I’ve been at a party once that someone was wearing a velvet jacket and I couldn’t be in the same room as them. Like literally, I couldn’t be next to them and I even asked them to take it off but obviously they didn’t (laughs). They were just like “what?” (laughs).

**Do you still go out regularly?**

Yeah, yeah. I still go out and everything. I think it’s just more a thing that I put on the back of my mind and it’s probably it gets easier as a get older but yeah. When I lived back home my mom…this is before she knew my massive issue and she had like a velvet settee and honestly I couldn’t even sit on it and I just didn’t go in the leaving room but yeah. She’s got rid of it now (laughs).

**Does the colour of the fabric influence the effect that certain fabrics have on you?**

Um…no. not to my knowledge. No.

**So if there is a fabric that you do like, and it has a colour that you particularly don’t like, if there is a colour that you don’t like, would it have an effect?**

I wouldn’t say so. No really.

**What is the right and wrong fabric for you to use?**

Polyester is like my favourite fabric. I wanted to bring my whole dressing gown but it’s really big. Because I think with polyester…

**Are you sure that that’s polyester? Because it’s much more different than the one I have here (experiment).**

Yeah, it says 100% polyester said on the label. But maybe this isn’t polyester, but I am sure it sais 100% polyester. What would you say this is then? Because last night I was figuring it out to explain it to you.

**It’s like wool, but it’s not. It’s really soft and the one I have here (experiment) is 100% polyester in the sense that it’s the material that make the tags so maybe your one is another use of polyester maybe? (Both laugh).**

Anyway, this is my favourite material whatever this is (laugh). So as soon as I go home, I put my dressing gown on. I literally live in my dressing gown. Because, I don’t know. I just feel like I can’t really feel this. Like it doesn’t have anything offensive about it.

**Tell me more about it.**

Like when I get my body wet, especially when I wash my hands, I literally cannot touch any materials afterwards. Like when I have a shows I cant even use a towel afterwards, like, I have to get straight into my dressing gown and I just cant touch anything for like an hour until like my hands are completely dry. So I moisturize them which is quite odd. But I choose to use this material because this literally the only thing that could touch when my hands are wet. All the other materials feel rough in a sense, or just kind of like heavy and they make my hands feel weird whereas this doesn’t.

**Is that because your skin is more sensitive when it’s wet if I understand correctly?**

Yes. My hand are way more sensitive when they are wet. Or even just washing my hands, afterwards I can’t touch anything for ages.

**So the right fabric is your dressing gown, the wrong one?**

Velvet.

**How does velvet affect you in your everyday life?**

It’s really hard to explain how it makes me feel but I can think of different fabrics and each would give me different sensations. Like, I am thinking…um…it’s odd. Because some fabrics make my teeth feel weird and if it think of another fabric it will just make my skin crawl and if I think of something else it different. But like, when I think of velvet or touch velvet like when I go shopping and I accidentally touch velvet it could actually make me feel it in my head. It’s like a sensation like at the front of my head. It’s like weird. It’s actually hard to touch it. It’s really odd.

**Does it cause you any anxiety?**

Yes. I will try to avoid it as much as I can. Like, all my friends know because it’s a massive issue so they know not to wear it around me. It’s like an anxious feeling. Like I will feel quite unsettled and it will only last for a few minutes and then I just think of something else and after a couple of minutes it’s okay.

**Are there any public places that you wished would use different fabric and why?**

Um… like the material that bus seats are made out of. Obviously some materials they use on the buses are different, but some of them, like they are really soft. Um…what is it? It’s not velvet but it’s kind of similar. I can’t stand that at all. No, I can’t stand that. Like if I get on the bus and the seats have that material, I literally just stand instead of sitting on it because it just makes me feel too uncomfortable. So yeah, if the busses used a different, nicer material. And they are always dirty and horrible and horrible colours. I can’t stand them yeah.

Are there any other places that you could think of?

Maybe like the material on swimming costumes. What is it called? Latex? S…

This one (pointing at spandex)? It’s Spandex.

Spandex. Yeah. I wouldn’t be able to wear because i don’t like the sensation of that either because if it could be sort of. Because you can’t find a lot of swimming costumes that aren’t made out of this material because it’s obviously waterproof so…yeah. Regarding my house, I chose it so I already made sure that none of the furniture was made out of anything I didn’t like. Like the uni is quite good. Like the library has all these different types of seats I can sit on and in that sense I don’t really have an issue with going there. I will always have somewhere to find a seat for me.

So now I would like you to touch each of the fabrics if you want, if you feel that you don’t want to touch a fabric you can always ask me and I’ll explain the fabric to you without you directly touching it. I would like you to explain your feeling and thoughts as you touch each of the fabrics and explain to me how these might affect your everyday life.

Satin: this one is fine because it’s light. Like I wouldn’t personally wear this but I don’t think there is anything bad about it. It kind of not airy enough. But if it was around me it wouldn’t cause me an issue. I think it’s too kind of like airy enough and I wouldn’t be able to wear it. Yeah. That’s not a bad one.

Denim. I don’t have an issue with this. I wear a lot of denim, I like it. I prefer the ones that are stretcher rather than this one. I would wear this as an oversized jacket, but I wouldn’t wear it as jeans or anything unless it was ‘stretchier’. I have a lot of oversized jackets which I like.

Hession: this just reminds me of a sack of potatoes (laughs). Do people wear this? (Laughs).

Maybe an avant-garde fashion show, but I don’t think so (laugh).

This is definitely something I wouldn’t be able to touch if my hands are wet because it’s like so rough and it’s something I wouldn’t be able to touch at all. I don’t know how to describe it. It’s like literally like, It causes me to seize up and cringe but it’s really had to put the feeling into words. It will make my hands hurt. Like, my hands will physically sting and it will make me highly anxious. I don’t know. It’s like I can’t process it. Yeah. It’s really hard to explain. But yeah, it actually physically make my hands hurt with certain materials. Even if I just washed my hands it’s like a really sort of rough feeling, so fabrics like this one are the worst ones so literally this (sleeping gown) the only thing that will be able to cope with because it is so soft. Yeah, I wouldn’t be able to deal with that one (laughs).

Cotton: it’s not too bad. But the only thing with this is that I can picturing this. Like if it was a table cloth or something I think I would have issues with like stuff moving over it if that makes sense. Like if I had a cup or something and wouldn’t be able to move the cup on it because of the texture of, you could hear the motion. I don’t like the sound of anything going over this material because it makes my head feel a little weird. Maybe I would wear it. Potentially not this one because it’s made out of cotton. But I think because this particular one is denser than others. I wouldn’t wear it like this, especially not the pattern (laughs).

Polyester: No, I don’t like this one. It’s horrible. That’s polyester? How is this (sleeping gown) and this (experimental polyester) the same thing? I don’t understand. That’s really weird. I don’t really understand fabrics, I just realised (laughs). But it makes sense if this (experimental polyester) is polyester because I have to cut all the tags out of my clothes because I hate the feeling of it and that’s probably why. So I don’t think this (sleeping gown) polyester and I don’t know what it is (laughs). But yeah, I don’t really like the feeling of that one (experimental polyester). That’s why I cut all my tags out because it feels like it’s digging into me and even if it’s not literally digging into my skin I can still feel it and it just makes my skin like ‘ugh’. It’s hard to explain but I just don’t like the sensation of it on my skin. I am not a fan of that. I don’t know how to describe it. It’s like when I stroke it I can feel like a sound which is really odd. I think that’s why I feel it so annoying on my skin. It’s odd. It’s like I can feel a sound, like ‘Z’.

Wool: I wouldn’t wear this type of wool. It’s quite like fuzzy feeling. It would annoy me in a sense. I wouldn’t be able to touch this either if my hands where wet. It’s too like, heavy. Yeah. No I don’t like that one. Not a fan.

Spandex: definitely don’t like that one. I would just feel trapped. I wouldn’t wear that as a swimming costume. You can get swimming costumes that it’s kind of that material but different. I am not sure what it’s called but I am sure it’s different, like tight and stretchy. I haven’t bought a new swimming costume in ages, but I remember the last one I had was when I went on holydays, so I had to just go out and look through different materials and choose one that I was okay with. This is too tight and makes you, well makes me (laugh) fell a little trapped and it would really irritate my skin and it would kind of, I don’t know. I wouldn’t feel free in it, so yeah. I wouldn’t wear something like that.

So, we will now talk about your favourite fabric and I would like you to explain to me why you chose it and how it influences you or affects you?

We don’t know what it is yet (laughs). The mysterious fabric (laughs). This is from my dressing gown but I wouldn’t bring my whole dressing gown (laughs). My dressing gown is like, my life saver because obviously I can just wear it in all of the house so don’t need to wear anything else. I don’t really like wearing clothes at all. It gives you a sensation when you wear it. It’s really soft and I can’t really feel it because it makes me feel like I am naked obviously I can’t be naked because of my housemates. My dressing gowns are also, it sounds weird but I don’t like the feeling of towels and I didn’t find a towel that could stand. I got one towel which is this kind of material but even then I don’t really use the motion of using a towel when I get out of the shower so I get straight into my dressing gown and it dries me but I have to wash it every couple of days because it goes really damp (laughs). I’ve got three of the same dressing gowns (laughs). I just use it for everything. It’s perfect. I even sleep in it because sometimes I don’t like the feeling of my cover and it’s literally my life saver. I don’t like my covers because I don’t know. The material is not offensive and I am okay with it. But sometimes it’s kind of, I don’t really like the inside of it and touch the covers. I will only get into the cover if I am really cold otherwise I just sleep on my bed an in my dressing gown and that’s fine for me. I use my dressing gown all the time. I think that’s why it’s my favourite material, because it doesn’t make me feel anything. It makes you feel like it’s warm and you can’t really feel the sensation of it on your skin and I don’t know. I just like it. It’s like my blanker. I just love my dressing gown. It’s super (laughs).

**Interview 8:**

**What does the type and texture of fabric mean to you?**

Mean to me. Um…I don’t know if I ever thought of them as having meaning necessarily. I don’t know. I brought, because obviously if you’ve got questions that you need to ask, but I brought this (showed personal object) because obviously I am 33, and I was only diagnosed early this year so obviously before that I didn’t know so I was trying to fit in and all that kind of stuff so I think probably my obsession with particular types of materials when I went to secondary school when I kind of realised that it wasn’t a thing that you do so you kind of had to stop kind of thing? Do you want to see now? I don’t know whether it’s going to…

**We will talk about your personal object afterwards, but it wouldn’t mind if you feel that it’s in the contexts of this.**

No, no, no, it’s fine because I don’t know how to answer the question because it implies that I thought about it but I am not sure whether I did.

**So the question is about if fabrics are important to you or not.**

Yeah, not the ‘meaning’ bit, yeah. It definitely affect me. So, I’ve always had to cut a lot of labels out of clothing. Not always, but if I notice them. Because sometimes I think that if I cut them out it actually makes it worst, because you can feel it like scratching. So sometimes I leave them unless I really notice them, but I always had a thing. I don’t enjoy being naked, but I also don’t enjoy lots of clothes. Like I hate when it gets to winter and you have to wear lots of layers and I suppose I am always aware of clothing and obviously the different fabrics which can be really annoying. So whatever I am doing, especially if I am with other people I suppose I am constantly aware to a large extent on what I am wearing so, you know if you feel sweaty or clothing is getting too tight because probably may have got a couple of pounds, I can literally notice it all the time so it could be frustrating because you are not focusing on what you should actually be focusing on doing if you see what I mean. So I wear a lot of dresses now because I like them but maybe also I suppose because they are a bit more comfortable because dresses are not as restrictive unless I have put a few pounds (laughs). That kind of thing. I always avoid like cheap fabrics, that’s ‘quotation marks cheap fabrics’ (laughs). You know, the type is that they feel really horrible and things. I don’t know does that makes sense?

**Yes, yes. Do you want to add something?**

I don’t know. Like I said, it’s the way it’s worded when you say ‘what it means to me’, because when you said ‘is it important’ that made more sense. The ‘importance’ makes more sense. So I suppose is always being aware of my own clothing and stuff which is not nice, it’s frustrating.

**Explain to me how the types and textures of fabrics affect your everyday life? Like the effect that they have on you, if they do.**

Well, mostly I would say negative, because they are distracting. Um…I mean I hate things that are like what you are wearing like a big thing around your neck and I have got a couple of jumpers like that but I want to wear them because it’s warm but they annoy me as well. Like I hate, I always hated things around my neck. It feels restrictive, even if they are big and baggy it feels quite restrictive. It’s weird because at the same time I like wearing big things. Do you know what I mean? I don’t like layers. It’s really annoying. So I don’t like layers, so I’d rather not wear this cardigan for instance as well as a dress but it’s cold and I have to. Um…and I always lose my train of thought. So, I like wearing my dresses and things but then as soon as I get home everything I wear at home is loose. Which is good my partner he also has autism and well he is autistic and he has ADD and like, my past partner would get annoyed because as soon as I get home I want my bra off and I know a lot of women I think do that but I always wear baggy old men’s, boyfriends big t-shirt and like big baggy doggy bottoms. So when I am home that’s literally is as soon as I get home it’s how I want to because I am much more comfortable like that and they are not as restrictive. But my past partner would really get annoyed with this and at least the one I got now he just knows that’s how I am the most comfortable whereas the past one was like “oh, but you dress up when you go out” and I was like “yeah because I am not going to wear my pyjamas out” (laughs). Yeah. What was the question again?

**It was about you explaining to me how the types and textures of fabrics affect you in your everyday life.**

Yeah, well, it depends on the fabric and there is obviously some fabrics that are really nice and then there are some that are horrible and they make. Um…I know you want to get onto specific ones at some point but my partner brought home literally like three massive boxes that were bigger than one of these tables and probably as big as this table of army blankets which are made of scratchy wool and I can’t touch them and he thinks it’s hilarious. There are certain things that make me make this weird, I call it my turkey gobble noise (laughs). So if I touch them, I’ll do it now but it’s voluntary but it’s sounds funny when it’s involuntary so it’s like “olulululu” (sound of turkey gobble) (laughs), like that, and I do that noise and he finds it hilarious because the noise is really funny but I can’t and hate touching the wool blanket. So there are certain materials that I would literally be, and I can’t really describe it. I just don’t want to touch them and even if I start thinking about them too much, which I don’t want to think about it now, but if I think about it too much then it makes me like “ugh” (cringe noise). Does that makes sense?

**Yes, yes.**

I know you are focusing on fabrics but like you say is tactile stuff and my partner he’s obviously kind of steaming he likes to sit down and do circular motions on my hand or my leg or something like that but if he does it for too long and I’ve always had that. If somebody like touches me for too long on one spot it’s unbearable and really horrible. It’s like, “don’t, you’ve have to stop doing that” cause it’s really uncomfortable situation and if that relates to why clothing if it’s in the same sport for too long it gets uncomfortable if that’s makes sense.

**So you said about the negatives, do fabrics have any positive effects on you?**

That’s more like my favourite fabric that you were talking about so should I talk about that now?

**Yes, but in regards to what effect it will have on you, what it would make you feel in a way?**

Well, so the ones that I enjoyed is satin until I went to school so from when I was a baby until I was in secondary school until I was like eleven that would have been a comfort and a security thing so like if I lost that which was a piece of blanket if I lost that or if I couldn’t find it um…I would be really disturbed. So I remember when I went to visit, because I lives with my grandparents, I didn’t live with my parents so I remember going to visit them on the weekend and I forgot to bring my blanket and it was horrible because I needed my granny to go or come and bring it and I was probably around eight so yeah, it was like a comfort thing in like feeling safe and when it used to be a big piece of blanket I used to hide under it and then it ended up being just a scrap and because I loved it so much it became like a scrap and ended up literally just being a scrappy bit. I would take it everywhere. So I suppose yeah. I can’t think of if there was like a sensory feeling that I can remember or that I relate to. I think it’s more like a mental thing and the comfort idea that you feel safe which is a little bit weird when you think of the piece of materials (laughs). I suppose I never thought about it. It’s nice but unless you get too attached to them because I remember I just had to tell myself when I had to stop taking it to school and stuff I would have had to tell myself really logically that you know “you can’t take your blanket to school, it’s not very grown-up thing to do” so you have to tell yourself even though it’s not very nice to not have it, if that makes sense.

**So, does the colour of the fabric influence the effect that it has on you?**

I don’t think so, because especially that satin piece, it was really, it was like a brown kind of grotty because I didn’t want it to go in the wash in case it got ruined in the washing machine. So with that it was about it was different, it was about the feel of it. If it was cold it would be better if it was cold and I always used to think it like, it sounds really weird but it smelled like ‘everywhere’ because I used to take it everywhere so t had this very distinct smell. It wasn’t disgusting considering that I didn’t like getting it washed because I looked after it. It had a very distinctive smell which I loved that made me feel happy I suppose.

**So the colour doesn’t have any effect?**

I don’t think so. I mean this colour particularly (Spandex sample) offends me slightly (laughs) because it’s really like in our face but I don’t think so. I mean it comes to wearing colours obviously I like them to match but sometimes they are all over the place different colours. I don’t know I never thought about it…colour. Yeah.

**What is the right and wrong fabric for you to use?**

Um…as I said. I hate wool especially if it’s like that really raw scratchy wool like those blankets are horrible. Say the question again?

**What is the right and wrong fabric for you to use?**

Wool. I can’t explain that cheap thing like what is that? (Pointing at spandex)

**That’s Spandex.**

So yeah. Kind of like this (spandex). You know when you get those cheap dresses from really cheap shops it’s that kind of material I suppose that just feels kind of restrictive and also it feels like. Um…you see like this (polyester) is quite nice. There are a bit too thick but they are quite like my blanket that I had. But this (spandex) if it was like in a dress like material isn’t very nice because it’s like makes you feel restricted but also very sweaty and things like that and it’s really uncomfortable. Well, I like cotton because they let you like, breathe and things. Again, they can be a little bit sweaty as well like if you are getting really hot or something. I am not that fussed about, but it feels warm to have that furry thing under my jacket. I would probably would be happier if it wasn’t in there but I got it because it’s warm so maybe like the weird fluffiness about it. I don’t know. I think mostly wool. I hate wool. This one (sample wool) is not too bad though but it’s not pleasant it’s like…I don’t know…it’s not. I should have brought those horrible blankets with me but then I wouldn’t be able to touch them so I would put someone to put them in the bag for me (laughs). The sound of it as well is horrible. Yeah. I am trying to think. Yeah, I don’t know.

**Are there any public placed that you wished would use a different fabric?**

Public places. Have you got any examples?

**Um…Like, the library, the student dorms, chairs, maybe some objects in town or something.**

Again, if it was like scratchy I would notice it besides catching something I am wearing. Um…I can’t think of anything. My sisters’ got a new velvety sofa now which you know if you rub it up the wrong way is weird but I don’t know. I don’t know if I ever thought about it before because usually I am so distracted of what I am wearing that I don’t know if I would like to notice it unless it’s really scratchy or wool. I don’t think I would, in public places. All I can think of is sits. That’s probably the only thing I can think of. In my head I am trying to picture them but in my head it’s just anything that is scratchy or catches on like your clothes. So I don’t know. Chairs are the only ones I could think of that I would touch and it would bother me I suppose. You know when you do conferences and you’ve got those boards that have that weird wool. That’s it because I don’t have to touch them all the time so it’s fine (laughs).

**So these are the fabrics and I would like you to touch each of the fabrics if you want to touch them, and tell me more about how it may affect you in your everyday life. If it affects you obviously.**

Like I said, 1 (satin) and 5 (polyester) are much more like my blanket so I quite like those because it’s like this coat, well I wear this one (pointing at one coat) and I brought this one (pointing at extra coat) and if I am wearing it sometimes I don’t realise I am doing it but I would be doing that, like from the inside rubbing my thumb and finger between the fabric kind of thing, like my blanket that I had. So those two feel like that. Number 1 is too thick though and you can’t do it (the rubbing) too much and like it doesn’t rub (laughs) whereas this (polyester) does quite nice. But it’s a mixture of things. It’s because it’s cold and I wouldn’t like it if it (polyester) was warm I don’t know why. It’s like doing that (rubbing) (laughs). It’s just a nice sensation I suppose because that’s (satin) is too thick for me to rub my fingers in between without friction so it’s not doing it enough whereas that one (polyester) does. I wouldn’t necessarily wear it (polyester). So it’s not about. I think it’s because it reminds me of my blanket which is that security thing. So the coldness…yeah.

**Denim:** like jeans material don’t really bother me too much apart when they are too restrictive.

Hession: This is okay, but I probably won’t be able to do that for too long because what is that made of?

**It’s Hession.**

It’s like those fine hears isn’t it. Yeah. I mean I wouldn’t be able to wear that (laughs). I am trying to think of where you see this because I have seen this in places.

**Potato sacks maybe?**

Yeah. I am sure must see it elsewhere as well.

**Like shopping bags that are recyclable?**

Maybe. Yes. Those cloth ones. But I wouldn’t be able to touch that for too long but yeah. It’s not particularly offensive to be honest. The weird noise and I would stop touching it. So yeah. It’s like I am not doing it for too long because it would cause a weird, unpleasant situation. So I would make that involuntary weird gobble, turkey gobble voice and have to stop whatever it is that is touching me or I am touching it. But sometimes I don’t even have to be touching it. Like, if I am, you know sometimes I go around with my partner and he’ll be like…sometimes is not just wool. It might be things like this (spandex) or that cheap velvety material and we would be walking round and my partner would be like, “oh this is really soft come touch this” and I am like, “I can’t even look at you touching it so I wouldn’t touch it myself” (laughs). So it gives me even before, because I can just recognise I suppose that a material will feel uncomfortable. So I am like “I am not going to do that because I am feeling uncomfortable and I am not even touching it yet”, kind of thing, and then he is like “but you should just touch it” and I am like “no, I don’t want to” (laughs). Yeah…so I would probably just avoid. I think I just learned over the years what I like and what I don’t like their sensation so I just avoid them.

**Cotton:** I like this one. It has green as well which is my favourite (laughs). It’s like curtain material. What is it?

**It’s cotton.**

Yeah, yeah. But would you have that in like a curtain material?

**I don’t know. It’s 100% cotton but it’s probably used in another way to make curtains I guess.**

**Polyester:** Yeah, I already like this one (laughs). It doesn’t feel like…you can tell. Like if you had a satin that was thinner I would probably like number one but It’s just too thick to do anything with. So I like polyester because you can rub it and you can still…because it’s not quite…you can feel like the fibres are quite coarse. So when I am rubbing my fingers is not quite the same. So I think my blanket was a lot finer in terms of the fibres because here you can actually feel the fibres when you rub it, so it’s not as nice if that makes sense. So yeah, if number 1 (satin) was thinner. I wouldn’t wear this though (polyester). The tags on clothes are a lot stiffer but this is not that stiff. So that wouldn’t be…I wouldn’t wear that. I tend to check out of what materials my clothes are made out of and see the percentages. If it’s mostly cotton I usually buy it but obviously it says polyester or elastin wouldn’t like that when the quantity is quite high. I don’t mind when it’s a little bit because it just means it’s got some stretch, but if it’s mostly one of those other materials I won’t buy it.

**Wool**: this is a lot finer so it’s not coarse. Um… (Laughs). It’s actually not that nice (laughs). I don’t know. I could probably wear it over something because it’s not that coarse but I think…actually it is. Um…I don’t know what it is. Because I can feel it has like silky fibres number five (polyester) but this one you can I don’t know. You can feel the fibres are different and obviously it’s like scratchy almost like little nods. I don’t like you number 6 (wool), go away (laughs).

**Spandex:** Number seven reminds me of that cheap dresses. I mean, that’s fine for swimming costume but I don’t like wearing those either. It’s because I don’t feel that comfortable being that exposed (laughs). But yeah. It doesn’t feel unpleasant like if I wasn’t wearing it but I suppose I could just do that (rubbing) almost. But then I wouldn’t like to wear it. I don’t know how to describe it. Sometimes they’ve got some little fabric in them, I don’t know. I am trying to imagine it in my head if I am wearing it. It may be too restrictive or just in my head I know I would probably be sweaty in it. So I am always aware of everything but I don’t want to feel that uncomfortable. I am just particularly aware if I am wearing something you know…because unfortunately we all sweat and stuff even when you wear the best clothes, so I am always aware “ok I am not going to wear that dress again” because you can tell. So if I am giving a talk or something and I just think that I don’t want people to see and I don’t want to feel that uncomfortable that I can feel that I am all sweaty and that kind of thing. This one doesn’t offend me, but number 6 (wool) offends me (laughs). Number three (hession) I won’t touch for long. Number seven (spandex) is okay but I wouldn’t wear it.

Now we will talk about your favourite fabric and I would like you to explain to me why you chose it and how it affects you?

I was thinking the other day what I can bring and why I love that fabric. So I brought photos of me when I was younger. So as I said, I was a foster child by my grandparents so it’s kind of like too slightly traumatic to be taken away from your parents and stuff and my sister was only a baby so as part of being a foster child you had to do like a photo album so you knew where you come from and stuff like that and I actually got a little story that was my favourite story that I called “the blanket” and it’s about when you go to bed and you’ve lost your blanket and then you find it again and that’s how important this little piece of material was. I am trying to find because I’ve got a picture. This is a picture of me as a baby with the whole blanket. I also used to suck on my thumb and used the blanket and it was another reason why I kind of had to not take it to school with me when I got older because I couldn’t stop my thumb at the same time. So yeah, this bit in middle…I had this massive blanket. Probably is not that big because I was a baby when I had it but the side bit if you can see is like this sort of material (polyester) and they are like folded over and sown to like keep the edges of the blanket. But when I started to get older it got really tatty and my granny when I was about four just cut one strip and kept me the one strip of the blanket and threw the rest of it away and I used to have nightmares and daydreams that the other piece of blanket was sitting on a rubbish tin waiting to be saved. It was so important to me and she just threw it away that I was like imagining it somewhere. So I had this little strips (showing photo). I had it into hospital with me, I had an operation when I was about nine and like I said, when it was this big when it was a big blanket I would be under it and I used to…um. So if you imagine that bit it was still folded over and like stitched on one side so you could have it doubled and you could do that sensation of the material by rubbing it on itself. I would like it if it was cold, so I would suck my thumb and then I would touch my nose because it felt soft and because it needed to be cold I would move down the strip so you would like, start when it got warm on one part of the strip I would move it along to have more of the coldness of the strip. It’s so weird talking about my blanket, I am 33 (laughs), because I haven’t talked about it for years or thought about it like that. But yes, you would move along the strip and you could tell it was so you only had the cold bit. So it smelled like lovely. It was just…you couldn’t describe it because it was “everywhere”. I always thought about it as everywhere I go, that’s what it smelled like. Like “everywhere”. I got upset…I don’t know why I was doing it, but I was like remember tying it under my arm and pulling it with my teeth to make a little nob and I was so upset because I tore a hole with my teeth in one end and things like that so yeah. Those sensations. It still actually had in part of it had…because if you imagine it had attached to the main bit which I can’t really remember how to describe that material. You don’t really have anything here that I would…maybe it was like a cotton or nice wool that It had been knitted. It’s kind of woolly to some extent. So if you can imagine if that’s the edge piece that I ended up keeping that I would call my blanket so the main bit is just sown in-between. So when my granny cut it off there is actually some of the main blanket inside. So there was like a thinner bit on one end and stuff like that and then when I stopped sucking my thumb I still have it but it’s at my granny’s’ house where I grew up. So when I stopped using it I got like a big normal like scarf, kind of like a wool scarf but not scratchy wool and the blanket should still be there folded in-between the scarf to keep it safe. So sometimes, I am thinking “oh, I hope it’s safe” (laugh). I am an adult now. But that particular fabric has so many important like elements to it like the sensation of it was nice and just made me feel comforted by the coldness and the silkiness and the smell of it. But I also used to imagine, because I was disturbed when the other bit was thrown away but then I would also while sleeping I would imagine that it came alive like a snake but like a nice snake because it was my blanket and I loved it. So like I imagined it being animated and like having feelings (laughs) all this sort of things. Yeah…um…even now I cannot describe the lovely smell that I liked about it but I can almost imagine it. I remember the smell but I cannot obviously describe it. In my head I can almost smell it and those sensations and stuff. My sister is also autistic but she is not diagnosed she had different. She would suck her thumb a little bit but she was more rocking and she says she still does it now as an adult. Mine was more like the fabric thing and we had different stimuli. I thought I had more, and I was trying to find more pictures of me with the blanket but those are the only two I could find. I don’t know if at the time it had emotional value but it does now if you think about it, but at the time it was just really comforting and all the sensations about it and stuff. That’s what I say, as an adult if I get a jacket like this one sometimes I just catch myself “oh you are doing that thing” (laughs). You are feeling it and stuff and makes you feel comfortable and stuff like that. I have replaced that kind of comfort, like sensation like with others. So I’ve noticed that as I’ve got older that I like and play with the silkiness of your nails and I do patterns quite regularly and sometimes I forget that I am doing it but it has to be in a certain pattern and the same rate that I would move along the blanket to the cold bit like you kind of do that because they are (nails) really soft and silky I suppose the nails aren’t they? So I notice that I do that quite a bit. So you can hide that more easily and it’s a bit more socially acceptable for somebody to sit there and play with their fingernails (laughs). You are thinking of more specific fabrics, but in relation to tactile issues that’s why my hair is like this because I hate my hair touching me, but this is still not short enough for me because it irritates if it touches my face and when I had long hair it would be up because as soon as it touched my neck I want it off so I ended up cutting it. I would like to have it all shaved off. I am going to, but I was trying not to upset my partner because he is also autistic and he’s got ADD so he is fine, btu I don’t want to like shock him. It’s not going to be bald but it’s going to be. Like, my hairdressers are so nervous about it and I a like “it’s really just hair”. It doesn’t bother me if it’s gone, it bothers me that it’s there because it’s irritating and you can like feel it and everything so…I know it’s not fabric but it’s still more like that tactile sensation of wanting it gone.

**It will be nice I believe.**

Yeah, my hairdressers told me that my hair is in a nice shape anyway so it’s fine (laugh). Like it wont look weird if I shaved it off. It doesn’t bother me. It’s not really about the appearance it’s more uncomfortable and annoying. So yeah…

**Interview 9:**

**What does the type and textures of fabrics mean to you?**

Mean to me?

**Is it important? Is it not important?**

Definitely with clothes shopping is important because I like soft fabrics like on my, or like silky stuff. Because I just see jumpers that look like they should be soft and you touch them and it’s not. And your life ‘ugh’ no (laughs). That’s not what I signed up for (laughs). So I do like really soft things because I want to be comfortable in it and like not itchy.

**So is it important to you?**

Yeah, I’d say so. Like with um…sort of clothes. I quite like cushions, like very soft, puffy cushions and like bedding and stuff should be soft and delicate. Things that are very plasticky, I am not very into those. Whether it will be fashion choice or the fact that it feels weird. Sometimes you have something’s that feel like a sweat suit or something. Ugh…it’s unpleasant.

**Why is the unpleasantness important though?**

Because when it’s unpleasant and I have to wear it I’ll be just continuously thinking about it and I’ll just be like ‘ugh’ the entire time (laughs). I like clothes that are soft and stuff so I don’t have to think about them later or I can fiddle with them but if it’s like a weird texture I’ll be like ugh. It makes me tense almost, like cringe. It’s like shivers and not pleasant ones. Because I think rough things are really horrid. Because I hate like polystyrene and I think is the most horrible texture ever like the little marshmallow things that you put in boxes. I don’t like how those feel and like nail files and sandpaper and stuff I really don’t like. It just makes me feel funny. Sorry I have a bit of a sore throat and if I sound weird.

**No, it’s fine, don’t worry about it. Do you want to add anything?**

Softer things are more comforting I thing. Like you feel more secure that you have something fluffier and softer whereas you feel exposed if is something very waxy or plasticky I suppose. It doesn’t make everyday things comfortable to do I think. Like if I had a really plasticky skirt I would just constantly be aware of the plastic skirt the whole time. Like, my day is not all the stuff to do. My day is like thinking what the hell the skirt is doing. So like, it’s distracting I could say. Like, I want non-distracting material (laughs). I could just be like ‘A! Nice, that’s good’. I don’t think I can pin-point an emotional element with that because I think I am just so used to I suppose. I am not sure how to describe it in terms of emotions. I wouldn’t say bad material makes me nervous. Maybe a little bit but it’s that it makes me uncomfortable and like, tense. So, I don’t like it.

**So, the next question is about you explaining to me how the types and textures of fabrics affect your everyday life?**

If you go back to the sandpaper and nail file feelings, I’ve got long nails because I hate nail files and the feeling is just ‘ugh’. It sounds funny but I am like ‘no!’ it’s the roughness of it. I think the only way to describe it is shivers down my spine or like ants or stuff if I am touching it because then I am just like ‘ugh’. I am taken ‘back’ by it. That’s like that with jumpers that look soft but are not naturally soft. Like my friends has a jumper that looks like the softest thing ever but it’s just not. It’s so coarse. Um…it doesn’t affect me too much but it definitely dictates my shopping. I will say that. I will spend a lot of shopping time touching materials until I find one that I like and I see um… if the design is nice after usually. Well, if I see there is a sign or I see something and I go “oh, I like that” but then I touch it and it’s horrible then I am just like “no, I am leaving that one, I am not buying that one” (laughs). Yeah. Soft things help me sleep and stuff definitely. And when I am ill I’d rather cuddle something soft but I think that’s more of a comfort thing for me and I am not sure if that’s a universal thing. Probably not.

**Can you tell me more about it?**

I am not sure. I have this really big plush, massive and when I am ill I just like to cuddle it and sit with it just like, pure comfort where is this squishy, comfy thing and I really don’t know why to be honest why I like it so much. I think it’s the fact that it’s manual or the fact that it can bend with my shape well whereas things that are more stiff are like “oh, I can’t press into it” because I like the feeling of smooth stuff though, like smooth metals and stuff or just things that are a bit too smooth or have grip so like, plasticky stuff is okay to an extent but I just wouldn’t want to wear it or I wouldn’t want it anywhere near my bed or something because that’s soft. I am not sure why it helps me sleep though. I think because when I was younger I was surrounded by fluffy things so I always liked toys and stuff. Bed is a comfortable place (laughs). It’s a place to turn-off for the day and stuff. I am sure stuff will come up.

**Does the colour of the fabric influence the effect that it has on you?**

Oh…that is a good question. It does. I hate obnoxiously bright stuff. Like, anything could hurt because I have eye sensitivity as well.

**Do you want me to close the lights?**

No, no, I am fine thank you.

**Are you sure?**

Yes. Don’t worry about it. It’s mainly auditory so I wear like headphones or earplugs in lessons and things but it’s like bright colours. I really don’t like obnoxiously bright colours because it hurts my eyes and I keep looking at that one (Spandex) (laughs). Because I consider my skin tone rather like dark. My skin tone is not dark but all my features are dark so I’ll dress more to meet my colours because it’s suits me better, so I just avoid them anyway. I don’t know it’s just very leery. Is that a good word for it? Leery? It’s like, ‘in your face’. I don’t like that because it can irritate my eyes quite easily. Like they feel um…I want to say scratchy but it’s more like a really soft itch in the back of my eye and like when I see something really-really bright I find like, um….pink is annoying but I think it’s worse with a really bright blue I think is the worst one. You know when your computer has malfunctions and you get a blue screen? That kind of blue. It’s like electric blue. That really hurts my eyes when I see it so it definitely lets me know that there is something wrong because I am just like “oh my god”. Highlighters are sort of okay, but I don’t use them a lot because my handwriting is very cursory as well so I just can’t use them because it just looks weird. You can’t highlight those writings, and so highlighters are not for me. It’s not too bad because I don’t find orange to be that bad for some reason. Like, the pink can hurt and depending on how bright the green is, green can hurt as well. Orange I don’t think is too bad, I just don’t like the colour (laughs). So definitely, the brighter the colour the worst it usually is in my opinion.

**What is the right and wrong fabric for you to use?**

To use in what sense?

To wear, to have in your house or wherever you encounter fabrics if that makes sense?

The softer the better usually. My go-to. Because I wear a lot of jumpers. I am a jumper maniac (laughs). Like this particular jumper I have like 3 of the same jumper in different shades and it’s like “what am I doing” (laughs) but I do like really soft ones that I’ve got and really fluffy cardigans and stuff. I go for fluffiness or just soft. It doesn’t have to be fluffy, it just needs to be nice to the touch like um…this jumper is probably not the best example because it’s little bit rough but it’s not rough enough to like annoy me and it’s soft enough that I like it as well. Like definitely more soft and something that does not restriction? Like when you move your thumb across it there will be no restriction from the fabric.

**Do you mean friction?**

Yeah, like no-friction, yeah. So I usually wear…I’ve washed this one a lot of times with a softener so probably no it’s softer than originally. But things just need to move across like on my arm (stroking arm). Also, when it’s fluffy, it gets warmer quicker which is what I like. I think things that aren’t soft enough don’t keep heat as much and so they are impractical at the end of the day and you are like “what is the point of you?” (Laughs). Like, I’ve got a, um… bomber jacket which is nice because I like the colours and stuff but the material is just s*** (laughs). It’s just like, this is thin and kind of coarse material that doesn’t stop any wind getting to you so it’s like, useless. So it’s literally just for you to look nice but it has no function and I rarely wear it. Which is quite understandable, because I like fluffy things. Yeah…definitely fluff I would say. Fluffy things are nice. I wear jeans a lot but I use fabric softener to make them a bit less rigid a lot of the time. Like if it’s not soft I’ll try making it soft and if I can’t make it soft then I don’t like it (laughs). So anything that’s too rough definitely and feels rubbery. Rubber, I don’t like. I don’t know why. Like, I remember going at a group in uni where there are a bunch of autistic women that we meet and have tea and chat about things and someone brought like a bunch of like stemming toys that are things to distract us with and one of them looked like a dog toy (laughs) but it was like, you put this rubber ring on your finger and there is a weird ball strings and you move it but ‘ugh’ it felt horrible! I felt really weird and smelled weird as well. Smell is important as well. If materials have strong smell then I get nauseous. So I don’t like leather that much especially in cars because I tend to get motion sick quite badly so if a car on top of that smells really bad I am just like “nope”. I can’t do that. I prefer fabric cars. I don’t like that you can stick to leather as well. So that’s a bit gross. Like if it’s a hot day and you have a leather sofa and like you are wearing shorts and go to get up after that you are like “au!” because it leaves half of your leg on the sofa because you are still stuck (laughs). Like, yeah. Strong sense is not nice. So the only examples I could think of is leather and rubber. Rubber is because it’s like soft but not soft at the same time I think. Like it’s waxy material, like, I don’t think it leaves a residue, but I think that when I touch something that is really rubber like that weird toy thing, it feels like it left something on my hand like a rubber texture on my finger tips and it’s horrible. Obviously you don’t get clothes from rubber which is quite convenient. Yeah. No. I am not a big fun of leather and I am a vegetarian so I don’t really buy it anyway…which is probably for the best. I also don’t like plastic things. Like rain coats. I don’t like those they are too waxy. That’s the only useful description I can give…waxy. Like, they are like stiff I think as well and there is like that stiff waxy things. I touch my fingers even though I am not touching something, that’s because I am trying to feel how it feels like (laughs). Like, artificial I suppose. So that artificial plasticky, waxy texture. I’d rather wear if it’s raining I’d rather wear a fabric coat any day just because anoraks is horrible. Clunky I’d say. It would sort of like feel like when you wear a normal coat and you can move around but when you are in that coat you are like in a position, stack (laugh). Like you can’t move in it. I don’t like that. I don’t move a lot but I want to know the choice is there. I think I am done now (laughs).

**Are there any public places that you wished used a different fabric? And why?**

Um…public places…I think like coffee shops tend to go for the more leather stuff and aesthetics and things like that. I would rather it be sort of nicer. Sorry I need to cough.

**Yeah, go ahead. That water is yours if you want some.**

Oh! That’s mine? Thank you.

**Yes, and the doughnuts (laughs).**

(Laughs). So bad. I don’t know what my throat is doing the last couple of days. It’s like “ugh” (laughs).

**(Laughs) maybe you are getting a little ill or something.**

Hope not. I really hope not (laughs). I am trying to think of things in public that I don’t like. The only thing that I can think of is the texture of um…you know when you go to a supermarket and I cant remember it’s name, but it’s like that things that you put your shopping on it and like it moves to the person on the checkout. Well, the material on that thing I don’t like. I don’t know why. I did work in a supermarket but I was at the café so I didn’t have to do that, but I had training down there and I had to go down there and touch it and I was like “nope”. It was like turn style so it’s couldn’t be a fluffy material but it was just a weird material because it needed that sort of smoothness and the grip to function which just felt horrible and I was just like “oh that’s not nice”. It’s the only thing that I can mainly think of but obviously as I said about cars but obviously not a lot of public transport uses leather. The one that I am aware of use the softer fabric which is not too bad. I am really trying to rack my brain with it but (laughs). I don’t like public places for other reasons but I don’t find them bad with fabrics.

**The university?**

Yeah, it’s fine. It’s okay. It has like smooth tables and relatively soft chairs and stuff. Like sometimes when it’s a bad material I can really get fixated on it. Like some tree leaves and plants they have that waxy feeling and I won’t probably stop touching it and I am like “how to feel like this?” (laughs). But I don’t believe there is anything that would like “this needs to change right now”. I don’t like shopping bags that much and I think they are trying to get rid of them anyway. You know the smaller ones. The little plasticky ones. I like the more big ones. Although I don’t like the feeling of them but I just like they are more functional. There is function over matter, but it’s feeling makes me feel weird. It’s lie “ugh”. You know the bags for life and you know, like the band that are synched together ones and I don’t like the material of those but is still use them and I don’t think that’s too rough. I think it’s those. I can’t think of anything. I mean if I do my memory maybe I can remember, but on the spot (laughs).

**You said things that are important to you and that’s perfect. Now we will move on to discussing these fabrics (pointing at sampled fabrics) and I would like you, if you want, to touch each of the fabrics and tell me little bit about them in terms of the feelings and thoughts that come into your mind and how they might affect you? If they do?**

From where do you want me to start with?

**With the first one and just go along if that’s okay.**

**Satin:** the top isn’t so bad, and I like the feeling of that. But this side (under) really horrible. Because I think with this side, the silkier side is a lot smoother and I like that I can like sort of move it down my fingers and its relatively easy and when I do it on my hand it just moves whereas the other side is a lot more “bluh”…rough. Very not my thing. This side is nicer. I don’t mind the other side. It’s not my favourite thing but I don’t mind it. The other side is nice.

**Denim:** this is denim. I like denim, I don’t mind denim. Because like, there is a little bit of tag on it but not enough to bother me. The first one (satin) felt like sandpaper whereas this one is denim. Denim is denim. I feel like this side (outer side) is bit nicer but I wouldn’t really feel it if it was on my leg apart if you would be touching the outside. Denim is not too bad. I wear denim a lot. I think it can sometimes have a bad odour but other than that its pretty good.

**Hession:** I felt funny touching that. That one felt weird. I don’t know. I don’t like this one (laughs). its like, I just get so hypersensitive to it. It’s like I almost feel every single stich in the 3^rd^ one. And like, it’s like bubbly and the fact that this fabric itself is so wired and coarse. Horrible texture. It’s like potato sack. Like if I do that (stroke it on hand) it’s just horrible. You can tell it more form my face I guess (laughs). Like, proper. I can’t stretch it. Rough. Not nice at all. I wouldn’t, I don’t get how people wear this. Function-wise it’s quite good because obviously milk cannot get through that. Some people wear straw hats or something and I am just like “how? Why would you want that on your head? Like on your scalp?” It seems really itchy and horrible. I feel itchy right now, like, touching it. Itchy feeling. Yeah, like. It’s not. Imagine a full suit made out of this that would be horrible.

**Cotton:** It’s not too bad. It reminds me of a shirt. Like, a tablecloth. Cotton. I don’t wear cotton. I wear more wool I think. It’s not too bad but let’s say I was at a shop I wouldn’t buy it unless I needed a shirt because obviously we are not at school like where there is uniform or that stuff anymore I haven’t needed to wear. Sort of shirts like this material. Because it feels like it would be like a button-up smart shirt. I don’t like that because they are not really comfortable all the time. Like, it’s not bad, but I wouldn’t wear it by choice. I’d wear it for necessity. It’s not the worst on. It’s not this one (Hession). It exists (laughs). I probably have t-shirts that are made of this. Is it more like a tea dress? For me it looks like it should be a dress but I don’t know if it is.

**It’s 100% cotton for sure but I don’t know if it was made for anything specific.**

Oh. Okay.

**Cotton:** it’s not that bad. It doesn’t feel weird when I do that (stroke it on hand). Like, I can move it fine. It’s not an offensive material whereas number 3 (Hession) and the underside of one (satin) are. Am I saying enough stuff?

Yeah, you’re doing great.

**Polyester:** That’s not what I thought it would be. That threw me off and when I touched it I was like “ah!” Um…because I thought it was plastic earlier. In my mind it was plastic or something and I touched it and I just went “ou!” because I am not wearing my glasses either so I didn’t see the piece and like, I wouldn’t wear this material but I am curious about it because it was not what I thought it would be before. It’s also nice to play with it. I wouldn’t probably wear it because I don’t want polyester stuff who does? I don’t think polyester stuff are that popular anymore? Polyester is like what my t-shirts are have a percentage out of it. I mean, it’s not bad but it threw me off because I touched it and I didn’t expect it to move under my hand. I was expecting it sort of retained itself and when I touched it it sort of “ploup” (laughs). it like, followed through. I was just like “oh, hello”. It’s nice though actually. It feels it would be cool to wear. I really don’t mind it, but it’s distracting because I am proper playing with it. It’s a nice distraction but it’s still a distractor. Because it makes a little noise. It just makes me curious more than anything. It’s not bad. Especially considering that it’s something I though I wouldn’t really like. So it’s not too bad. That’s really nice. I think after a while you would be just sick of wearing it as a shirt and stuff because it’s a bit too smooth. I think t-shirts should deffinately be a percentage of this because it kind of reminds me of a proper like silk 90’s and the fact that it moves so easily. Polyester fabrics are so packed so unlike this one (hession) is really loose but this one is really dense. It actually feels quite tight and silk is really tight as well. This is a relatively cheap silk (satin) and I think that’s what throws me off a bit and that the underside is “bluh”. The top is not too bad because I think like proper expensive silk is really –really lovely if you like, get your hands on it sort of thing. It’s cool and usually I am after warm stuff but in the summer if it’s like a summer night I’d want something that is cool and like proper silk. Like, number 5 is not too bad (polyester).

**Wool**: also what I haven’t thought (laughs). Maybe I am thinking I am not wearing wool then? Is this pure wool?

Yes, but I guess it’s a different type of wool? More dense? But it’s 100% wool.

I think I prefer mixtures, because this is the sort of material that I mean when I say “when you think that something would be quite nice to touch and it’s not” (laughs). I was actually looking forwards to this one because I thought it would be quite nice but it is a bit rough. The loose fibres in it, I can feel everyone and I think that’s what makes it uncomfortable for me because it’s not like easy to move. Like, my fingers need to go over the fabric. That’s not as nice as I wanted it to be (laughs) it’s probably 2^nd^ least favourite after number 3 (Hession). I feel I like, if a mixture of 4 (cotton), 5 (polyester) and 6 (wool) is probably the best option to get out of each thing perhaps. Because I can see how this be soft, but in this state it’s not and it’s very soar. It feels itchy as well. Like, if I put it over my, like, the top of my hand its scratchy and it would be horrible to wear it as a jumper and I will probably be aware of it all day rubbing against your skin. It would probably make me irritated after a while. Hm… (Laughs).Not my favourite. Not at all. It actually proves that I don’t know what wool actually is.

**Spandex:** number 7 (spandex) is the leery one. Oh god! Oh! I spoke too soon. Oh that is horrific. Oh! I know it’s funny and I know it’s swimsuit stuff (laughs) it’s just like. It’s like that rubbery texture and it actually smells. I think that that’s how I notice that I don’t like this kid’s swimsuit or something. That is horrible. Maybe…it’s obviously made to use into water. This is actually two materials! Which is interesting because the material that your skin actually touches it’s not too bad and it kind of feels like a softer version of the underside of number one (Satin). It’s the other waterproof side that I don’t like. It’s like where the smell comes from and it’s the…I do like that it’s stretchy but like…it’s not pleasant to touch. It’s like function over comfort sort of thing for me at least. It’s not that it’s restrictive because I can move fine and stuff. It’s just like I said before it’s like it’s leaving something that it’s not. I feel that it should be leaving something. Like, if I go like that (rub on hand), it should be bright pink now. Like it left something. I think it’s just my mind telling me that I just don’t like it (laughs). It’s like, the inside side is fine. Well it’s not fine. It’s like…definitely the smell because the smell really puts me off quite a lot. Like if there was a way to make it senseless then maybe. Because like that rubbery-ness that I don’t like. Try to get my words out. Like. If the scent was gone, then it probably wouldn’t be as bad. It is like the feeling of, like, this waxy, rubbery ness within the fibres that puts me off as well. It just feels like it sticks to your hand, even if it’s not. It feels like it’s leaving a layer of whatever this rubber stuff was. I don’t know how else to describe it.

**Okay, you did great. Now we will go on to talk about your favourite fabric and I would like you to tell me why you chose this in the first place, and how it influences you?**

Hm…I think it might be either number.

**I am sorry. I didn’t phrase the question correctly maybe. Your favourite fabric. The one that you brought with you.**

Oh, it’s like, the proper fluffy stuff?

**Yes, yes.**

Like um…that material (showed leggings that have fluff underneath. It looks physically like fur and I like that material. I don’t know what the synthetic fur. I like that sort of material that I like the most. Like, big, fluffy jumpers and I like the fact that it’s inside my leggings. Toys, and other fluffy things like that and big cushions. I think it’s synthetic fur. I think. Because sometimes you can find relativey scratchy ones, but I find it’s usually um…stuff that doesn’t contact with the skin a lot, like those fluffy key chains they are usually quite rough. But stuff that are meant to touch your skin, like jampers and things like that are fine. I think. I try to think what its called. There is synthetic fur and there is like really really soft PJ’ which look fuzzy but it’s not quite fuzzy, it’s just really soft. I really like that.

**Why do you chose these to be your favourite ones?**

They make me feel like warm and cosy a lot of the time. They don’t irritate my arm or anything and they don’t make me think about it too much. I like being comfortable than looking good it think (laughs). I’d rather just wear what makes me comfortable and for me being comfortable means wearing a lot fluffy things. So I get more enjoyment out of fashion stuff that are fluffy. It’s like…definitely like. If you had to name the fabrics I think it would be yarn and do you know yarn? Like, there are some balls of yarn that you can get that are lovely and soft. It looks like Kashmir. I love Kashmir and that’s a really nice feeling because I have no restriction and it’s soft and doesn’t have odour and I really stuff like that. I think if it doesn’t makes me aware of its presents and doesn’t make me aware of it then it’s a pleasant experience and I tend to like it.

**Tell me more about it.**

Because I fiddle a lot and necklaces and trim of my short. And if I do fiddle I want that to be something that I actually feels nice. So if I was wearing something like that (wool) it would be like grotesque (laughs). I would be like “oh. No, no!” (Laughs). Wouldn’t want that. I might fiddle and I don’t want to be aware of my fiddling because of that coarse, horrible fabric would do that whereas I would rather just split sort of thing and don’t pay mind to it. Yeah…because like, I get distracted easily I think as well. It’s that easy. So if the material is wrong it would be all I can think about probably and it would be very destructive and probably in any situation. Like, I would not listen to people probably because I would be like “why are you so soar? Why are you such a horrible material?” (Laughs) and I don’t want to be distracted by this thing. That’s why I pick softer ones. Because I sometimes you are doing your own thing and once you touch a bad material you are just stressed on that material oh my god. It’s like, there is more focus on that bad material.

**Interview 10:**

**What does the type or textures of fabrics mean to you?**

I suppose it’s mostly just a way of my everyday life like what you wear really. I mean, it’s easier when you are an adult because you can choose your own clothes rather than…say parents actually put you into dresses or something so…yeah I mean it’s more of the choice of clothing rather than my environment.

**Are fabrics important to you or not?**

I suppose it’s important in terms of which one is more has more comfort otherwise it’s not something I think about like too much.

**Can you elaborate more, in terms of its importance?**

I suppose it is a bit important at times meaning that a fabrics can be comforting and say, with anxiety that can help a lot you know, just to calm you down and stuff…and just… I suppose it’s important to dress generally comforting and I suppose coping with other sensory load at times. Like, going to a fabric that you actually like, instead and try to calm myself down?

**What happens when it affects you differently?**

Hm…I mean, in that case it’s just trying to remove myself from the fabric if possible and usually it’s like itchy tags and stuff of nylon. I am kind of buying everything cotton now and I’ve always done that since I was younger and everything. So, in terms of everyday fabrics and stuff, it’s usually labels that affect me the most.

**So the labels are important you said. Why?**

Kind of yeah. Just like, especially when you can feel it poking into your skin and stuff and it can be really adjusting to feel. Really uncomfortable. And you know, when you wear several layers and the label like folds or something and your clothes all are tack. So I regularly remove them a lot quite often so…I mean sometimes you can’t always know which way the clothes actually go around or something but generally that’s what I do.

**Okay. So explain to me how the types and textures of fabrics affect your everyday life?**

I suppose it’s a case of how I feel in the day and like sometimes I can cope with certain fabrics but other times it can be overwhelming like, I know that silk bedding, that’s quite overwhelming. It does feel nice, but it can become overwhelming quite quickly and just like velvet is not one that I like. It’s like, what I said before about the tags really. I mean, most of the clothes I am wearing are cotton anyway so it’s not too bad.

**What kind of effect does it have on you specifically?**

It depends on the fabric but sometimes it just causes discomfort and obviously I can remove it from me or something. I mean, sometimes you wake up more like, sensory sensitive anyway and then other days no, so it just kind of depends really when that happens. Let’s say, in terms of wearing layers on a day to day basis and like wearing like a hood and a coat or something that would really frustrate me so, you know, I have to wear a hoodie or a cardigan with a coat rather than having that hoodie pushed down as well. Also things around my neck and the feeling of suffocation sometimes and so, say, in high school like, top buttons and stuff depending on the kind of layers you wear because sometimes it could be right up and like give you some space.

**Do you want anything to add?**

I don’t really think so.

**Do you have any specific areas in your life that they are mostly affected?**

I’d say fancy dress you know like, say around, Halloween stuff because of what the costumes are made out of very scratchy material underneath and even though they’d have like something underneath that’s like trying to ease that, you can still feel it and stuff and just… Mostly the fancy dresses and the more formal-wear you know, when your parents would put you in formal dresses and stuff, so…which I would not really put in these situations. So, I think that’s like the main case of when I was younger since now I have more choice over what I can wear and stuff so, not too bad. So like, clothes stores or something and I like to feel the material obviously as I go on and you know, when you notice a nice material and you think “oh I want to feel that” then that’s quite nice and to wear your dressing gowns because I like the weight and I have to have all fleece pyjamas and stuff so that’s quite nice material.

**Does the colour of the fabric influence the effect that certain fabrics have on you?**

I suppose if they are particularly bright, then obviously, because I am quite eye sensitive anyway, so generally I choose quite plain colours just because it’s easier to match in the first place. It’s also just a more comfortable. Hm…especially around summer time and my mum would be like “oh why don’t you wear this bright thing, or buy this bright thing from the shop” and it’s just not for me (laughs).

**So the colour does have an effect on you?**

Yes.

**So what is the right and wrong fabric for you to use?**

I suppose like fabrics and stuff like cotton, fleece, silk, but not in large quantities and bad fabrics could be like, velvet and I suppose like metal, like an itchy metal in a way. Just…or I suppose at times kind of denser materials let’s say.

**Why is that?**

I suppose is that fact that like, stuff like cotton and silk is more like compactly made and other stuff are pulled apart sometimes so it can agitate my skin a little bit like when the light touches my skin through the material.

**Oh, do you mean, when the material is denser the better?**

Yes. Just in terms of denim and stuff I’ve never really likes that because of the feeling and obviously I don’t really wear jeans a lot and stuff or at least not often and when I was younger I never liked tights because you know, especially during the removal and stuff. Kind of that tight feeling. I don’t really like it. I am kind of okay with it now but when I was younger I’d prefer more loose clothing and stuff like that.

**Are there any public places that you wished would use a different fabric?**

Not to me. Mostly I am hyper sensitive to light and sound so it’s not really like the fabric. I suppose in terms of like reflecting light from materials then yes, in terms of trying to reduce that glare but otherwise I can’t really think of much in terms of that. I suppose when you have couches or something in a public place and they are kind of made of felt or something it would be nicer if you know, where made of a different material instead. But I wouldn’t say it bothers me that much really as in other senses instead.

**Okay. Now, I would like you to touch if you want, each of the fabrics and tell me your feeling and thoughts about each one and explain to me how these might affect you in your everyday life if you had to encounter them?**

**Satin:** number one (satin) is okay. I wouldn’t say it’s my favourite kind of material especially like the under layer of it. Otherwise it’s not too bad and it’s not something you encounter that often so let’s say no it wouldn’t bother me that match really. Never been a really big fan of satin. It’s not something I would wear that often because it doesn’t really feel particularly nice to me. I mean, it feels like it should be really soft but when you touch it it’s just it doesn’t look as it seems really. It’s kind of a bit rougher than you expect. I imagined it to be a bit more like silk and it’s not so that could be a bit odd.

**Denim:** this is not too bad. I mean, generally I wouldn’t mind, but I wouldn’t wear it myself often, but occasionally I wouldn’t mind to come into contact with it. Denim is a hit or miss really. It depends on the day I suppose.

**Hession:** number 3 (Hession) isn’t particularly nice (laughs). Yeah…it’s quite scratchy and just generally if I were to touch that then I’d remove my hands away from it quite soon especially on like tables and stuff like that. Yeah…I wouldn’t touch that.

**Cotton:** it just reminds me of general like blinds and stuff. Like kitchen. Again, it’s okay but it’s not something I would use for comfort or generally wear. With clothes, cotton is quite softer because you know, I use softener for my clothes a lot. So, I suppose it takes away that kind of feel from it.

**Polyester:** number 5 (polyester) is okay. It’s quite good. I mean, it’s possibly okay but I think it will start to get overwhelming soon but I don’t think it’s a material I’d wear often in like due to cold and stuff. I mean, I am generally okay with that. It feels quite comforting in small quantities I suppose.

**Wool:** this is awful (laughs). Yeah…I can’t say I like that one at all. Reminds me of like, brillo pads those cleaning things for like ovens and staff, you know that metal bars, that kind of rough material a bit between felt and sandpaper and it’s just…yeah…I can’t say I like it. Yeah, it’s kind of something I can make a tingle in my hand when it touches so yeah, that’s a no.

**What it would make you feel if you touched it?**

I suppose it depends on the environment I am surrounded in and how bad I would be feeling that day then it could potentially cause sensory overload and I mean…it’s something I’d generally avoid.

**Spandex:** it doesn’t feel too bad. I mean, it’s mostly like, reminds me of like costumes when I was younger. That tight material and while I don’t mind the material but kind of like, the tightness of it can be overwhelming. So, I don’t mind the feel of it and the first contact but say like, if it’s stretched across my skin or something and then generally I would be bit adverse to that kidn of material. I mean, I am not exactly a sports person so, it’s not something that I come into contact with that often. If I had to, then I would wear it but probably I wouldn’t go too far particularly on time because of the kind of like suffocating kind of feeling of it. I mean, it’s not like very breathable material. I wear kind of looser material like cotton, as I mentioned before so, yeah…generally I wouldn’t wear that.

**So now we are going to talk about your favourite fabric, and I would like you to explain to me why you chose this one, and how does it affect or influence you in your everyday life?**

What I brought is a bit like fleece. I don’t really know what kind of material this is. It’s just a scarf but it could be quite comforting to wear so yeah, as I am walking around and then I stroke the material could be quite comforting and I generally like stuff like that. I mean I have a cat at home and she is quite nice to stroke when I am feeling particularly anxious on that kind sort of level. It’s kind of nice to seek that kind of material really rather than avoid it. It’s just generally more softer in a way that that’s okay. It’s not really like silk, it has like more layered texture to it rather than that kind of flat feel so I think it makes it a bit nicer to experience. I mean, I don’t know if you know what kind of material this is?

**It is soft! I don’t know actually. Maybe it’s cotton but not like the sample one. Maybe it’s less manufactured?**

Yeah, it’s like fleece on like pyjamas I wear a lot and stuff so, that kind of soft layered material. It’s not kind of one layered material. It’s kind of pulled apart and that.

**Does it have any importance to you?**

Not really no. No, I don’t really think so. I mean, it’s something I felt in the shop as something soft and I just liked it and obviously I felt it. I mean, it’s particularly nice when I am anxious and all that and to like, stroke it or something in like public it’s kind of more acceptable way of calming yourself and it’s kind of like, a subtle way of calming yourself down really. Yeah
